# Supplementary material for: EstablishINg the best STEp-up treatments for children with uncontrolled asthma despite INhaled corticosteroids (EINSTEIN): protocol for a systematic review, network meta-analysis and cost-effectiveness analysis using individual participant data (IPD)
Source: BMJ Open. 2021 Feb 5;11(2):e040528. doi: 10.1136/bmjopen-2020-040528 (PMC7925932; doi:10.1136/bmjopen-2020-040528)
Supplement: Supplementary data [file bmjopen-2020-040528supp001.pdf]

EstablishINg the best STEp-up treatments for children with uncontrolled asthma despite INhaled corticosteroids (EINSTEIN): Protocol for a systematic review, network meta-analysis and cost effectiveness analysis using individual participant data (IPD)

Sofia Cividini[1], Ian Sinha[2], Sarah Donegan[1], Michelle Maden[3], Giovanna Culeddu[4], Katie Rose[2], Olive Fulton[*], Dyfrig A. Hughes[4], Stephen Turner[5], Catrin Tudur Smith[1]

[1]Department of Biostatistics, University of Liverpool, Liverpool, UK
[2]Alder Hey Children's Foundation NHS Trust, Liverpool, UK
[3]Liverpool Reviews and Implementation Group (LRIG), University of Liverpool, Liverpool, UK
[4]Centre for Health Economics & Medicines Evaluation, Bangor University, Bangor, UK
[5]University Court of the University of Aberdeen, Aberdeen, UK
[*]Patients Representative, Liverpool, UK

Supplementary File 1. Example MEDLINE (OVID) search

1 exp Asthma/
2 asthma.ti,ab.
3 1 or 2
4 exp Infant/
5 infant*.ti,ab.
6 infancy.ti,ab.
7 newborn*.ti,ab.
8 baby*.ti,ab.
9 babies.ti,ab.
10 neonat*.ti,ab.
11 preterm*.ti,ab.
12 prematur*.ti,ab.
13 postmatur*.ti,ab.
14 exp child/
15 child*.ti,ab.
16 schoolchild*.ti,ab.
17 "school age*".ti,ab.
18 preschool*.ti,ab.
19 kid.ti,ab.
20 kids.ti,ab.
21 toddler*.ti,ab.
22 exp Adolescent/
23 adoles*.ti,ab.
24 teen*.ti,ab.
25 boy*.ti,ab.
26 girl*.ti,ab.
27 exp Minors/
28 minor*.ti,ab.
29 exp Puberty/
30 pubert*.ti,ab.
31 pubescen*.ti,ab.
32 prepubescen*.ti,ab.
33 exp Pediatrics/
34 paediatric*.ti,ab.
35 pediatric*.ti,ab.

36 exp Schools/
37 "nursery school*".ti,ab.
38 kindergar*.ti,ab.
39 "primary school*".ti,ab.
40 "secondary school*".ti,ab.
41 "elementary school*".ti,ab.
42 "high school*".ti,ab.
43 highschool*.ti,ab.
44 or/4-43
45 "inhaled corticosteroid*".mp.
46 ICS.mp.
47 exp Beclomethasone/
48 beclomethasone.mp.
49 "beclomethasone dipropionate".mp.
50 becotide.mp.
51 clenil.mp.
52 ciclesonide.mp.
53 "clenil modulite".mp.
54 exp Fluticasone/
55 "fluticasone propionate".mp.
56 fluticasone.mp.
57 flixotide.mp.
58 exp Budesonide/
59 budesonide.mp.
60 Mometasone Furoate/
61 mometasone.mp.
62 exp Adrenergic beta-Agonists/
63 "long acting beta-2 agonist*".mp.
64 "long acting beta2 agonist*".mp.
65 LABA.mp.
66 exp Formoterol Fumarate/
67 formoterol.mp.
68 Oxis.mp.
69 "fluticasone furoate".mp.
70 exp Salmeterol Xinafoate/
71 salmeterol.mp.
72 serevent.mp.
73 vilanterol.mp.
74 exp Leukotriene Antagonists/
75 "leukotriene receptor antagonist*".mp.
76 LTRA.mp.
77 zafirlukast.mp.
78 montelukast.mp.
79 exp Theophylline/
80 theophylline.mp.
81 Tiotropium.mp.
82 spiriva.mp.
83 Symbicort.mp.
84 Seretide.mp.
85 flutiform.mp.
86 relvar.mp.
87 or/45-86
88 Clinical Trial.pt.

89 Randomized Controlled Trial.pt.
90 exp Random Allocation/
91 exp Single-Blind Method/
92 exp Double-Blind Method/
93 exp Cross-Over Studies/
94 exp Placebos/
95 RCT.ti,ab.
96 Random*.ti,ab.
97 "Single blind*".ti,ab.
98 "Double blind*".ti,ab.
99 "triple blind*".ti,ab.
100 placebo*.ti,ab.
101 or/88-100
102 3 and 44 and 87 and 101
103 limit 102 to ed=20140701-20190911
104 limit 103 to english language
105 (case reports or editorial or letter).pt.
106 104 not 105
