# Supplementary material for: EstablishINg the best STEp-up treatments for children with uncontrolled asthma despite INhaled corticosteroids (EINSTEIN): protocol for a systematic review, network meta-analysis and cost-effectiveness analysis using individual participant data (IPD)
Source: BMJ Open. 2021 Feb 5;11(2):e040528. doi: 10.1136/bmjopen-2020-040528 (PMC7925932; doi:10.1136/bmjopen-2020-040528)
Supplement: Author's manuscript [file bmjopen-2020-040528.draft_revisions.pdf]

BMJ Open is committed to open peer review. As part of this commitment we make the peer review history of every article we publish publicly available.

When an article is published we post the peer reviewers' comments and the authors' responses online. We also post the versions of the paper that were used during peer review. These are the versions that the peer review comments apply to.

The versions of the paper that follow are the versions that were submitted during the peer review process. They are not the versions of record or the final published versions. They should not be cited or distributed as the published version of this manuscript.

BMJ Open is an open access journal and the full, final, typeset and author-corrected version of record of the manuscript is available on our site with no access controls, subscription charges or pay-per-view fees (http://bmjopen.bmj.com).

If you have any questions on BMJ Open's open peer review process please email info.bmjopen@bmj.com

BMJ Open

EstablishINg the best STEp-up treatments for children with uncontrolled asthma despite INhaled corticosteroids (EINSTEIN): Protocol for a systematic review, network meta-analysis and cost-effectiveness analysis using individual participant data (IPD)

Journal:	*BMJ Open*
Manuscript ID	bmjopen-2020-040528
Article Type:	Protocol
Date Submitted by the Author:	15-May-2020

Complete List of Authors:	Cividini, Sofia; University of Liverpool, Biostatistics<br>Sinha, Ian; Alder Hey Children's NHS Foundation Trust<br>Donegan, Sarah; University of Liverpool, Biostatistics<br>Maden, Michelle; University of Liverpool, Liverpool Reviews and Implementation Group<br>Rose, Katie; Alder Hey Children's NHS Foundation Trust<br>Fulton, Olive; Patients Representative<br>Hughes, Dyfrig; Bangor University, Centre for Health Economics and Medicines Evaluation<br>Turner, Stephen; University Court of the University of Aberdeen, Department of Child Health<br>Tudur-Smith, Catrin; University of Liverpool, Biostatistics

Keywords:	STATISTICS & RESEARCH METHODS, Asthma < THORACIC MEDICINE, Epidemiology < THORACIC MEDICINE, PAEDIATRICS

SCHOLARONE™
Manuscripts

I, the Submitting Author has the right to grant and does grant on behalf of all authors of the Work (as defined in the below author licence), an exclusive licence and/or a non-exclusive licence for contributions from authors who are: i) UK Crown employees; ii) where BMJ has agreed a CC-BY licence shall apply, and/or iii) in accordance with the terms applicable for US Federal Government officers or employees acting as part of their official duties; on a worldwide, perpetual, irrevocable, royalty-free basis to BMJ Publishing Group Ltd ("BMJ") its licensees and where the relevant Journal is co-owned by BMJ to the co-owners of the Journal, to publish the Work in this journal and any other BMJ products and to exploit all rights, as set out in our licence.

The Submitting Author accepts and understands that any supply made under these terms is made by BMJ to the Submitting Author unless you are acting as an employee on behalf of your employer or a postgraduate student of an affiliated institution which is paying any applicable article publishing charge ("APC") for Open Access articles. Where the Submitting Author wishes to make the Work available on an Open Access basis (and intends to pay the relevant APC), the terms of reuse of such Open Access shall be governed by a Creative Commons licence – details of these licences and which Creative Commons licence will apply to this Work are set out in our licence referred to above.

Other than as permitted in any relevant BMJ Author's Self Archiving Policies, I confirm this Work has not been accepted for publication elsewhere, is not being considered for publication elsewhere and does not duplicate material already published. I confirm all authors consent to publication of this Work and authorise the granting of this licence.

For peer review only

EstablishINg the best STEp-up treatments for children with uncontrolled asthma despite INhaled corticosteroids (EINSTEIN): Protocol for a systematic review, network meta-analysis and cost-effectiveness analysis using individual participant data (IPD)

Sofia Cividini[1], Ian Sinha[2§], Sarah Donegan[1], Michelle Maden[3], Katie Rose[2], Olive Fulton[*], Dyfrig A. Hughes[4], Stephen Turner[5], Catrin Tudur Smith[1§**]

[1]Department of Biostatistics, University of Liverpool, Liverpool, UK
Catrin Tudur Smith – Institute of Translational Medicine, Department of Biostatistics, University of Liverpool, Crown Street, Liverpool L693BX, UK – cat1@liverpool.ac.uk – Tel. +44 (0)151 794 4059/+44 (0)151 794 9754 – ORCID: https://orcid.org/0000-0003-3051-1445
Sofia Cividini – Sofia.Cividini@liverpool.ac.uk – ORCID: https://orcid.org/0000-0003-2705-9224
Sarah Donegan – Sarah.Donegan@liverpool.ac.uk – ORCID: https://orcid.org/0000-0003-1709-2290
[2]Alder Hey Children's Foundation NHS Trust, Liverpool, UK
Ian Sinha – Ian.Sinha@alderhey.nhs.uk
[3]Liverpool Reviews and Implementation Group (LRIG), University of Liverpool, Liverpool, UK
Michelle Maden – mmaden@liverpool.ac.uk – ORCID: https://orcid.org/0000-0003-4419-6343
[4]Centre for Health Economics & Medicines Evaluation, Bangor University, Bangor, UK
Dyfrig Hughes – d.a.hughes@bangor.ac.uk – ORCID: https://orcid.org/0000-0001-8247-7459
[5]University Court of the University of Aberdeen, Aberdeen, UK
Stephen Turner – s.w.turner@abdn.ac.uk – ORCID: https://orcid.org/0000-0001-8393-5060
[*]Patients Representative, Liverpool, UK
Olive Fulton – olive.fulton@gmail.com

Running head: The EINSTEIN Study Protocol (IPD-NMA)

[**]Correspondence to Professor Catrin Tudur Smith; cat1@liverpool.ac.uk

[§]CTS and IS are joint senior authors

ABSTRACT

Introduction

Asthma affects millions of children worldwide – 1.1 million children in the UK. Asthma symptoms cannot be cured but can be palliated with low dose inhaled corticosteroids (ICS) in the majority of individuals. Treatment with a low dose ICS, however, fails to control asthma symptoms in around 10-15% of children and this places the individual at increased risk for an asthma attack. At present, there is no clear preferred treatment option for a child whose asthma is not controlled by low dose ICS, and international guidelines currently recommend at least three treatment options. Herein we propose a systematic review and individual participant data network meta-analysis (IPD-NMA) aimed to synthesise all available published and unpublished evidence from randomised controlled

For peer review only

trials (RCTs) to establish the clinical effectiveness of pharmacological treatments in children and adolescents with uncontrolled asthma on ICS and help to make evidence-informed treatment choices. This will be used to parameterise a Markov-based economic model to assess the cost-effectiveness of alternative treatment options in order to inform decisions in the context of drug formularies and clinical guidelines.

Methods and analysis

We will search in MEDLINE, the Cochrane Library, the Cochrane Central Register of Controlled Trials (CENTRAL), Embase, NICE Technology Appraisals, and the NIHR HTA series for RCTs of interventions in patients with uncontrolled asthma on ICS. All studies where children and adolescents were eligible for inclusion will be considered, and authors or sponsors will be contacted to request IPD on patients aged <18. The reference lists of existing clinical guidelines, along with included studies and relevant reviews, will be checked to identify further relevant studies. Unpublished studies will be located by searching across a range of clinical trial registries, including internal trial registers for pharmaceutical companies. All studies will be appraised for inclusion against predefined inclusion and exclusion criteria by two independent reviewers with disagreements resolved through discussion with a third reviewer. We will perform an IPD-NMA – eventually supplemented with aggregate data for the RCTs without IPD – to establish both the probability that a treatment is best and the probability that a particular treatment is most likely to be effective for a specific profile of the patient. The IPD-NMA will be performed for each outcome variable within a Bayesian framework, using the WinBUGS software. Also, potential patient-level characteristics that may modify treatment effects will be explored, which represents one of the strengths of this study.

Ethics and dissemination

The University of Liverpool committee on Research Ethics has confirmed that ethics review is not required. The dissemination plan consists of publishing the results in an open-access medical journal, a plain-language summary available for parents and children, dissemination via local,

For peer review only

national, and international meetings and conferences, and the Press Offices of our HEIs. A synopsis of results will be disseminated to NICE and BTS/SIGN as highly relevant to future clinical guideline updates.

PROSPERO registration number: CRD42019127599 – This project is funded by the NIHR Health Technology Assessment Programme (project number 16/110/16).

Keywords inhaled corticosteroids ▪ long-acting β_2-agonists ▪ leukotriene receptor antagonist ▪ theophylline ▪ exacerbation ▪ asthma control ▪ hospital admissions ▪ adolescents

Strengths and limitations of this study

- This study will be the most comprehensive synthesis of interventional studies of children and adolescents with uncontrolled asthma on low dose ICS (indicatively, 200 and 400 µg BUD/day).
- It will be able to establish the most clinically and cost-effective step-up therapy for asthma, also considering those treatments where no head-to-head comparison exists, providing evidence to make informed treatment choices.
- It will be able to evaluate the influence of patient-level characteristics on treatment effect.
- It will provide an assessment of the incremental cost per quality-adjusted life-year gained of treatment options, from the perspective of the NHS and personal social services.
- Individual participant data may not be available from all eligible RCTs and integration with aggregate data may be needed.

INTRODUCTION

Rationale

Recent epidemiological data show that an always-increasing number of people of all age group worldwide, among that millions of children and adolescents, are affected by asthma with a notable social and economic burden for health systems.[1] Asthma remains a common medical condition affecting over one million children in the UK, one of the highest prevalence rate worldwide.[2, 3] Asthma is characterised by symptoms of wheeze, breathlessness, chest-tightness, cough and can

For peer review only

87 affect the child's quality of life by limiting daily activities and causing acute attacks. In the UK, it
88 was estimated that one child is admitted to the hospital every 20 minutes because of an asthma
89 attack.[2]

90 The British Guideline[4, 5] on the management of asthma recommends that, following a
91 diagnosis of asthma in a child, a stepwise approach to treatment should be taken. The initial level of
92 treatment is a low dose inhaled corticosteroid to prevent symptoms plus a short acting β_2-
93 adrenoceptor agonist (SABA) to relieve symptoms. However, treatment with a low dose ICS fails to
94 control asthma symptoms in around 10-15% of children.[6] If asthma remains uncontrolled, a series
95 of further steps are followed consisting of a treatment step-up by including add-on preventer
96 therapies such as long-acting β_2-adrenoceptor agonists (LABA) or leukotriene receptor antagonists
97 (LTRA), increasing the dose of ICS, or adding sustained-release (SR) theophylline.[1, 4, 5]

98 Choosing the best step-up treatment thus becomes a crucial decision to prevent occurring of
99 exacerbations and to avoid poor asthma control, also improving the quality of life of patients and
100 their families and optimising the use of NHS resources.[7-12] At present, no clear preferential
101 option for initial step-up exists, and the decision-making process about treatment choices mainly
102 bases on clinicians' preferences and not on evidence-based practice. Moreover, there is substantial
103 heterogeneity among individuals in the treatment response.[13, 14]

104 A recent Cochrane review[15] comprising of 33 trials with 6,381 children demonstrated that
105 adding LABAs to ICSs was not associated with a significant decrease in the rate of exacerbations
106 requiring systemic steroids but was superior for improving lung function (e.g., FEV_1 and PEF),
107 compared with the same or higher doses of ICS. No statistically significant group difference in
108 hospital admissions was found. Notwithstanding, because of a trend towards increased risk of
109 hospital admission with LABA independently of ICS dose, the authors advised further monitoring
110 of treatment.[15]

111 Another Cochrane review[16] published in 2013 found that adding LABAs to ICSs was
112 associated with no statistically significant reduction neither of the use of oral corticosteroids as

For peer review only

rescue nor hospital admissions compared to the same or an increased dose of ICS. Evidence was based only on 4 trials with 559 children and adolescents having mild to moderate asthma.[16] The authors cautioned that the lack of paediatric trials, the absence of data on pre-schoolers, and the variability in the reporting of relevant clinical outcomes was a limit for firm conclusions regarding this comparison.[16]

Two network meta-analyses (NMAs), analysing published RCT-level aggregate data for children with uncontrolled asthma, have already been conducted,[17, 18] but the evidence from these analyses has important limitations. The considerable variation in, and the incomplete reporting of outcome measurements across RCTs prevented a formal NMA and assessment of relative efficacies of treatments in one NMA of 23 RCTs (4,129 patients).[18] In 2015, Zhao et al.[17] conducted a formal NMA consisting of 35 RCTs with 12,010 children. These authors suggested that combined ICS and LABA treatments were most effective in preventing exacerbations and that treatments with medium- or high-dose ICS, combined ICS/LTRA, and low-dose ICS seem to be equally effective.[17] However, the analysis has limitations as the authors excluded 70 relevant RCTs because publications of these RCTs did not provide data about 'exacerbations' or 'symptom-free days'. Outcome reporting bias[19] is, therefore, a severe threat to the validity of their results if the excluded studies had selectively reported results based on the statistical significance of their findings. Moreover, a series of other issues make the interpretation and generalisability of the results of this NMA difficult. There is a lack of complete overlapping of the included studies compared to the previous Cochrane reviews[15, 16] and previous planned NMA[18] – only 7 RCTs are in common across the two NMAs.[17, 18] Two other weak points are represented by the lack of analyses focussing on outcomes of importance and relevance to patients and the lack of analyses comparing different drugs, doses, and types of inhalation devices within ICS, LABA, and LTRA classes.

Several randomised clinical trials (RCTs) have compared two alternative step-up options head-to-head, but only a few individual trials have analysed more than two classes head-to-head.

For peer review only - http://bmjopen.bmj.com/site/about/guidelines.xhtml

The BADGER trial[14] randomised 182 children and adolescents with uncontrolled asthma on fluticasone to receive each of the following therapies: 'ICS step-up', 'LABA step-up', and 'LTRA step-up', obtaining differential responses to every step-up treatment. 'LABA step-up' showed significantly more likely to provide the 'best' response; yet, many patients better replied with 'ICS or LTRA step-up' suggesting that for some patients, these treatments may be preferred.[14] Overall, results from BADGER trial were heterogeneous, and a 16-week follow-up may be no sufficient.

The MASCOT trial[20] randomised children aged 6-14 to fluticasone plus placebo, fluticasone/salmeterol plus placebo, and fluticasone plus montelukast. However, this RCT failed to recruit an adequate number of patients due to significant organisational challenges, issues of accessing patients from primary care, and changes in prescribing habits during the trial. These challenges limit the potential success of similar future clinical trials, and recommendations have been made for the use of alternative study designs[20, 21] to compare alternative step-up treatments.

There is thus an urgent need for a novel approach to be taken to synthesise all of the available evidence using robust, unbiased methods, also including unpublished data. A key strength of the EINSTEIN analysis will be the assessment of individual patient-level characteristics in modifying the patient's response to treatment. No study has previously considered this aspect, although it represents a crucial clinical step in evaluating treatment efficacy. Furthermore, an economic analysis of the treatment options will provide evidence on their cost-effectiveness from the perspective of the National Health Service in the UK. Results from the EINSTEIN study will provide clinicians and patients with accessible, high-quality, patient-relevant information to help make evidence-informed treatment choices. Earlier identification of the best step-up treatment for a particular child could have a significant impact on children’s lives with more extensive benefits to society and the NHS.

Objectives

For peer review only

Our research objective is to establish the best treatment option for patients younger than 18 whose asthma symptoms are uncontrolled by low-medium dose ICS. To this goal, the EINSTEIN study will identify and synthesise all available evidence from RCTs to establish the effectiveness of pharmacological treatments in children and adolescents aged <18 having poor asthma control on ICS. The study will also investigate the modifiers of treatment effect (e.g., age, ethnicity, asthmatic phenotype) to optimise a targeted therapy and maximise patients' informed choice of treatment. The economic analysis will estimate the cost-effectiveness of treatments in terms of their incremental costs per quality-adjusted life-year gained.

Specifically, we aim to:

1. Conduct a systematic review to identify relevant RCTs of treatment with children and adolescents whose asthma remains uncontrolled despite ICS use.
2. Collect individual participant data (IPD) from all eligible trials.
3. Carry out a network meta-analysis of IPD to identify the most effective treatment.
4. Identify modifiers of treatment effect to establish which patients respond better to each treatment.
5. Develop an economic model to estimate the incremental cost per quality-adjusted life-year gained of treatment options, from the perspective of the NHS and personal social services
6. Identify remaining uncertainties formulating recommendations to inform priorities aimed at future research.
7. Disseminate findings.

METHODS

The PRISMA-P guidelines (Preferred Reporting Items for Systematic Reviews and Meta-Analysis Protocols)[22] have guided the drafting of this protocol.

Eligibility criteria

For peer review only

For inclusion in the EINSTEIN study, we will select studies according to the criteria outlined below.

Study designs

We will include parallel and cross-over randomised controlled trials (RCTs), of any duration, using any level of blinding and comparing at least one of the health technologies of interest. All RCTs meeting our inclusion criteria will be included irrespective of the outcomes reported in the publication, to reduce the potential for outcome reporting bias.

Participants

We will focus on children (<12 years) and adolescents (12-17 years) of any ethnicity with poor asthma control on ICS as defined by the study authors. We will include studies with mixed age groups in which children and adolescents were eligible because we will contact authors concerning specific data on patients aged <18. Patients aged ≥18, with stable asthma as defined by the study authors, and using an asthma medication other than ICS, or an ICS with an add-on active (e.g., ICS/LABA) before entering the clinical trial will not be considered. Participants using low-medium dose ICS will be eligible for inclusion.

Interventions

We will consider as eligible all studies where children and adolescents were randomised to at least one of the following treatments:

▪ Inhaled Corticosteroids (ICS), alone or in combination with other treatments – beclometasone dipropionate; ciclesonide; fluticasone propionate; fluticasone furoate; budesonide; mometasone.

▪ Long-acting β_2-agonists (LABA) – formoterol; salmeterol; vilanterol.

▪ Leukotriene receptor antagonists (LTRA) – zafirlukast; montelukast.

▪ Theophylline.

Any dose of treatment and any inhaler devices – pressurised metered-dose inhaler, dry powder inhaler, combination inhaler – will be considered. Presence or absence of spacer will be noted.

For peer review only

For peer review only - http://bmjopen.bmj.com/site/about/guidelines.xhtml

We will not consider studies with participants randomised to any asthma medication other than those listed in the inclusion criteria, or pharmaceutical formulations other than inhalation formulation (e.g., tablets).

Comparators

For both primary and secondary outcomes – if not specified otherwise – patient outcomes will be compared at two levels, namely among:

1. Every class of treatment – ICS (alone or with add-on actives), LABA, LTRA, theophylline, and placebo. For ICS, we will distinguish between low, medium, and high doses as defined in GINA – Global Initiative for Asthma 2019[1] (Table 1 and Figure 1).

Table 1 Estimated clinical comparability daily doses (µg) of Inhaled Corticosteroids (ICS)

**≤ 5-year-old (Children)**			
**Drug**	**Low Dose**	**Medium Dose**	**High Dose**
Beclomethasone dipropionate (HFA)	100 (≥5 years)	N.A.	N.A.
Budesonide nebulised	500 (≥1 year)	N.A.	N.A.
Budesonide pMDI + spacer	N.A.	N.A.	N.A.
Fluticasone propionate (HFA)	50 (≥4 years)	N.A.	N.A.
Mometasone furoate	110 (≥4 years)	N.A.	N.A.
Ciclesonide	N.A.	N.A.	N.A.
**6-11-year-old (Children)**			
**Drug**	**Low Dose**	**Medium Dose**	**High Dose**
Beclomethasone dipropionate (CFC)	100-200	>200-400	>400
Beclomethasone dipropionate (HFA)	50-100	>100-200	>200
Budesonide (DPI)	100-200	>200-400	>400
Budesonide (nebules)	250-500	>500-1000	>1000
Ciclesonide	80	>80-160	>160
Fluticasone furoate (DPI)	N.A.	N.A.	N.A.
Fluticasone propionate (DPI)	100-200	>200-400	>400
Fluticasone propionate (HFA)	100-200	>200-500	>500
Mometasone furoate	110	≥220-<440	≥440
**≥ 12-year-old (Adults and adolescents)**			
**Drug**	**Low Dose**	**Medium Dose**	**High Dose**
Beclomethasone dipropionate (CFC)	200-500	>500-1000	>1000
Beclomethasone dipropionate (HFA)	100-200	>200-400	>400
Budesonide (DPI)	200-400	>400-800	>800
Ciclesonide (HFA)	80-160	>160-320	>320
Fluticasone furoate (DPI)	100	N.A.	200
Fluticasone propionate (DPI)	100-250	>250-500	>500
Fluticasone propionate (HFA)	100-250	>250-500	>500
Mometasone furoate	110-220	>220-440	>440

Table adapted from GINA 2019.[1]
CFC = chlorofluorocarbon propellant (no longer used; included for comparison with older literature)

For peer review only

DPI = dry powder inhaler
HFA = hydrofluoroalkane propellant
N.A. = not applicable

2. Every drugs within every class of treatment – beclometasone dipropionate; ciclesonide; fluticasone propionate; fluticasone furoate; budesonide; mometasone; formoterol; salmeterol; vilanterol; zafirlukast; montelukast; theophylline; (placebo).

Moreover, we will explore complex hierarchical models that consider both classes of treatments and actives with interactions of covariates. Healthy controls will not be considered as an appropriate reference group.

Outcomes

Review outcomes will include the outcomes identified as important to healthcare practitioners and patients during the development of a core outcome set for trials in children with asthma.[23, 24]

Primary outcomes:

- Exacerbations as defined by ERS/ATS.[24]
- Asthma control measured by a validated test (e.g., ACT,[25] ACQ,[26-29] other).

Secondary outcomes:

- Symptoms/symptom score
- Quality of Life (QoL)
- Mortality – although rare, this is an important outcome for parents
- Physiological outcomes (e.g., FEV_1, bronchial responsiveness)
- Adverse effects (AEs) – including growth and withdrawals due to adverse effects
- Hospital admissions
- Cost, resource use, and utility outcomes to inform the economic model

Timing

Studies will be selected for inclusion independently of the length of follow-up of outcomes.

Setting

For peer review only

There will be no restrictions by type of setting.

Language

We will include only articles reported in the English language.

Information sources and search strategy

Our search strategy will identify published and unpublished studies and builds upon previously published search strategies for aggregate data network meta-analyses[17, 18] and Cochrane reviews.[15, 16, 30-32] (see Supplementary File 1; for example, MEDLINE search). The search strategies will run from 2014 onwards only. From inception to 2013, we will consider as exhaustive the searches already carried out for the meta-analyses and Cochrane reviews above mentioned,[15-18, 30-32] and we will retrieve all the corresponding studies.

We will search MEDLINE, the Cochrane Library, the Cochrane Central Register of Controlled Trials (CENTRAL), Embase, NICE Technology Appraisals, and the NIHR HTA series using relevant search terms. We will also scan the reference list of included studies and relevant reviews along with the reference lists of existing clinical guidelines such as British Guideline[4, 5] and GINA.[1]

For unpublished studies, we will search a range of clinical trial registries enclosed inside the WHO 'International Clinical Trials Registry Platform' search portal (including clinicaltrials.gov and ISRCTN) and conference abstracts (e.g., European Respiratory Society; American Thoracic Society).

We will also search internal clinical trial registers for pharmaceutical companies that manufacture health technologies of interest (e.g., GSK, AstraZeneca, Novartis, Merck).

We will limit the search to English language articles, and we will verify that RCTs have included participants aged <18 as a subset.

STUDY RECORDS

Data management

For peer review only

Studies identified for screening of abstracts and full-text articles will be managed within Covidence (https://www.covidence.org/home), a systematic review management tool.

Selection process

All studies will be screened and assessed for inclusion by two independent reviewers (SC and KR) with disagreements resolved through discussion with a third reviewer (IS). To minimise the impact of selective outcome reporting, we will include all RCTs meeting our inclusion criteria irrespectively of the outcome(s) reported in the publication. The authors will thus be contacted to ask for information and the IPD for all outcomes relevant to the review.

Data collection process

The first author, or sponsor, of each included trial will be approached and asked to supply anonymised IPD, metadata, and relevant documentation[33] (protocol and blank case report forms) from the respective trial, either through direct contact, or through data sharing portals such as clinicalstudydatarequest.com, where relevant.

Before starting the quantitative synthesis, we will conduct a set of standard quality and consistency checks on the provided data. This will involve cross-checks by re-analysing IPD against previously published results to highlight inconsistencies or possible errors. Any queries will be raised with the original trialists wherever possible. Data will be cleaned and standardised to allow pooling and subsequent analyses of the data.

Data items

The data requested from each clinical trial will include at least:

- Baseline characteristics – age; sex; ethnicity; eczema; height; weight; baseline severity; baseline Peak Expiratory Flow Rate (PEFR); Forced Expiratory Volume in one second (FEV_1).
- The date of randomisation and dates of follow-up visits/interval between randomisation and follow up.
- Treatment details including inhalation device and dose.

For peer review only

- Adherence data if available.
- Data for the review outcomes along with details of their definitions and measurement tools used – symptoms; exacerbations; asthma control; mortality; quality of life; growth; physiological outcomes: Peak Expiratory Flow Rate (PEFR) and Forced Expiratory Volume in one second (FEV_1); hospital admissions.
- Cost, resource use, and utility outcomes to inform the economic model.

We will use data from the intention-to-treat (ITT) patients.

Outcomes and prioritisation

Primary outcomes

The primary outcomes of the study are exacerbations and loss of/change in asthma control measured by a validated test.

1. Exacerbations are defined as in ERS/ATS,[24] namely "*events characterized by a change from the patient's previous status*". We will define exacerbations as every event requiring the use of oral corticosteroids (OCs), the need for unscheduled visits with general practitioners (GPs), or at the emergency department (ED), and hospitalisation.
2. Based on the definition reported in ERS/ATS,[24] asthma control corresponds to "*the extent to which the various manifestations of asthma have been reduced or removed by treatment*".

Secondary outcomes

The secondary outcomes of the study are as follows.

1. Symptoms/symptom score – The mode to report symptoms or symptom score may be different across studies. We will distinguish between daytime and night-time symptoms, whatever they are, also considering their intensity.
2. Quality of life – QoL may differently have been measured across the studies. We will use the most frequent scale as a model to transform the remaining data using, e.g., established conversion tables.

For peer review only

3. Mortality – This variable may be reported in different ways across studies. It will always be converted to a binary variable.
4. Physiological measurements – FEV_1 in L/s and PEFR%. After consulting clinicians, we will decide if considering the full longitudinal profile (multiple measures) or establishing a clinically relevant time-point.
5. Adverse events – These may differ across the studies. We will consider relevant AEs, such as cardiac and neuropsychiatric disorders, infections, pneumonia, behavioural difficulty, growth, and diabetes.
6. Hospital admissions – This variable may be reported in different ways across studies. We will look for evidence such as dates of hospitalisation, adverse events indicating admission occurred, and variables directly reporting hospitalisation, between the randomisation visit and the end of the follow-up.

Economic outcomes

7. The primary economic outcome will be the incremental cost per quality-adjusted life-year (QALY) gained. This will be estimated from data on:
    a. Hospital admissions
    b. Other items resource use, costs, and utilities – These will be based on purposive searches of the economic and medical literature.

Risk of bias in individual studies

Two independent reviewers will use the Cochrane Risk of Bias tool[34] to record risk of bias concerning randomisation method, allocation concealment, blinding, incomplete outcome data, and selective reporting. Disagreements will be resolved through discussion with a third reviewer.

DATA

Synthesis

For peer review only

For every separate outcome, we will create a NMA diagram displaying the number of studies and patients for each treatment comparison within the network.

We will fit a Bayesian hierarchical meta-analysis model – supplemented with aggregate data if necessary – to synthesise the available IPD. We will thus estimate the relative treatment effect – odds ratio (OR) for categorical data and difference in means for continuous data – and credibility interval for every pair-wise comparison based on direct evidence. We will assess the homogeneity assumption by comparing the Device Information Criterion (DIC) of fixed-effect and random-effect models and observing the between trial variance. The forest plots, chi-square test for heterogeneity, and I^2 statistic will be examined to assess the evidence of heterogeneity within each pair-wise meta-analysis based on direct evidence.

We will conduct a NMA for every outcome within a Bayesian framework with the WinBUGS software with the Goodness-of-fit assessed by calculating the posterior mean residual deviance with DIC used as a basis for model comparison. Correlation between treatment effects from multi-arm trials will be appropriately accounted for. From the NMA, we can estimate the relative treatment effect for every pair-wise comparison irrespective of they have been compared directly in an RCT as well as calculating the probability that each treatment is the best, or worst, for a given outcome. In random-effects NMA models, we will check the conventional assumption that between-trial variance is the same for each comparison, by fitting pair-wise models based on direct evidence and assessing whether the variance is similar for each comparison. If the assumption appears unrealistic, we will explore other variance structures for the NMA model.

In a NMA, the indirect comparisons are not protected by randomisation, as for direct comparisons. So, they may be affected by confounding due to differences among clinical trials.[35] The validity of a NMA thus depends on the critical assumption of transitivity namely, to verify whether the probability of having been able to have given any of the treatment in the network to any patient in the network was equally likely.[35] We will check this assumption by comparing the inclusion/exclusion criteria of trials to make a judgement about whether patients, trial protocols,

doses, administration procedures, and so on are similar in the modes that might modify treatment effect. We will use model fit and selection statistics to informally assess whether inconsistency is evident along with a formal analysis using a 'node-splitting' approach.[36, 37]

We will explore potential patient-level characteristics that may modify treatment effects using hierarchical models with interaction effects between treatment and specific covariates. Initially, based on direct evidence, and subsequently in a NMA of the IPD, with aggregate data if IPD is unavailable.[38-41] The effect of covariates will be separated within and between trials. The underlying consistency assumption of these models will also be explored. Patient-level characteristics of interest include age, gender, ethnicity, eczema status, asthmatic phenotype, baseline %PEFR, and asthma severity. A literature search will be conducted to identify any further potential characteristics to explore.

We will fit a NMA model to compare compounds within each class and separate models to compare the different classes of treatments. Furthermore, complex hierarchical models that account for both classes and compounds with covariate interactions will be explored.

We will undertake sensitivity analyses for investigating the impact of missing IPD at the level of results and conclusions of NMA and for exploring the robustness of results to different priors in the Bayesian analyses. If sufficient data are available, sensitivity analyses that adjust for levels of adherence will be conducted to assess the robustness of results obtained from the primary analysis on the ITT patients.

Meta-bias(es)

To minimise the impact of selective outcome reporting, we will include all RCTs meeting our inclusion criteria irrespectively of the outcome(s) reported in the publication. For analyses containing 10 or more trials – due to the low power of the assessments for analyses containing small numbers of trials – we will investigate publication bias (and other selection bias/small study effects) by using funnel plots and appropriate statistical tests for funnel plot asymmetry.

For peer review only

Confidence in cumulative evidence

We will assess the quality of evidence for all outcomes across the domains of risk of bias, consistency, directness, precision, and publication bias according to GRADE (Grading of Recommendations Assessment, Development and Evaluation).

Economic analysis

The economic analysis will be conducted using standard methods,[42] based on the most robust data available and reported according to the CHEERS statement.[43] Cost-effectiveness will be estimated from the perspective of the NHS and Personal Social Services in line with NICE guidance[44] and based on an economic model that considers health outcomes, resource use, costs, and health utilities to estimate the incremental cost per quality-adjusted life-year (QALY) gained of each treatment.

The parameters needed to populate the models will include outcomes estimated by the NMA of the IPD (e.g., efficacy and safety parameters) and other parameters (e.g., utilities, resource use, and the long-term costs of care) that will require searching of evidence beyond the studies included in the NMA. These will be sourced from a purposive review of the literature, and by using specialist databases (e.g., NHS EED for resource use parameters, and the Tufts CEA registry for utility parameters) where necessary. Unit cost data will be derived from standard national sources (e.g., NHS reference costs[45] and the British National Formulary[46]).

A Markov model will be necessary for longer term extrapolation of costs and outcomes, to allow for any differential impacts of treatments over time, and for transition of patients among the 5 steps of treatment.[47] The model will evaluate costs and outcomes over the lifetime of the patient cohorts: costs and benefits in future years will be discounted at an annual rate of 3.5% and varied between 0% and 6% in sensitivity analysis. Results from the model will be reported as incremental cost per QALY gained (ICERs) and compared with the NICE threshold range of £20,000 to £30,000 per QALY.

For peer review only

Uncertainties in all parameter inputs will be accounted for in the analysis by including parametric distributions for each point estimate, supplemented by expert opinion, where the evidence is not sufficiently detailed. This will enable probabilistic sensitivity analyses to be performed based on sampling from distributions using Monte Carlo simulation. The NMA estimates relative effects jointly, and the full joint distribution of these effects will be used in the economic model in order to preserve correlation.[48] Uncertainty in the optimally cost-effective treatment will be represented by cost-effectiveness acceptability curves (CEACs),[49] which present the probability that each drug is the most cost-effective at a given threshold of cost effectiveness. Where the IPD-NMA indicates that patient characteristics modify treatment effects, a stratified approach will be used to assess cost-effectiveness in particular patient sub-group(s). Standard techniques (e.g., extreme value scenarios) will be used to ensure the internal validity of the model.

We will conduct a value of information analysis to inform future research priorities, e.g., whether more short-term efficacy trials are needed, or more long-term follow-up, or more data on the utilities or costs to reduce decision uncertainty.[50] The expected value of perfect information (EVPI) and the expected value of perfect parameter information (EVPPI) will be calculated on both per-patient and population levels using the Sheffield Accelerated Value of Information approximation[51] to facilitate computation effort.

Authors' contributions

CTS is the guarantor. CTS and IS conceived the study. CTS, SD, ST, DH, OF and IS drafted the original version of the protocol, and SC drafted this manuscript. All authors contributed to the development of the selection and data extraction criteria and the strategy of risk of bias assessment. MM developed the search strategy. CTS and SD provided statistical expertise. IS and ST provided clinical expertise. DH contributed to the section on health economics. OF contributed to coordinating the group of patients and parents and developing the plain-language summary. All authors read, provided feedback, and approved the final manuscript.

For peer review only

Funding statement

This work was supported by NIHR HTA grant number 16/110/16. The funder had no role in the protocol development. The views expressed are those of the authors and not necessarily those of the NHS, the NIHR or the Department of Health.

Competing interests statement

The authors declare not to have conflicts of interest.

REFERENCES

1 Global Initiative for Asthma (GINA). Global Strategy for Asthma Management and Prevention, 2019. www.ginasthma.org

2 Asthma UK. Access date: February 2020. https://www.asthma.org.uk/about/media/facts-and-statistics/

3 NHS England. Access date: February 2020. https://www.england.nhs.uk/childhood-asthma/

4 British Thoracic Society SIGN. British guideline on the management of asthma. Access Date: February 2020. https://www.brit-thoracic.org.uk/quality-improvement/guidelines/asthma/

5 Asthma: diagnosis, monitoring and chronic asthma management. Access Date: April 2020. https://www.nice.org.uk/guidance/ng80

6 Turner S, Thomas M, von Ziegenweidt J, et al. Prescribing trends in asthma: a longitudinal observational study. *Arch Dis Child* 2009;94(1):16–22. http://dx.doi.org/10.1136/adc.2008.140681

7 Schmier JK, Manjunath R, Halpern MT, et al. The impact of inadequately controlled asthma in urban children on quality of life and productivity. *Ann Allergy Asthma Immunol* 2007;98(3):245–51. https://doi.org/10.1016/S1081-1206(10)60713-2

8 Fuhrman C, Dubus JC, Marguet C, et al. Hospitalizations for asthma in children are linked to undertreatment and insufficient asthma education. *J Asthma* 2011;48(6):565–71. https://doi.org/10.3109/02770903.2011.580031

For peer review only

9 Sala KA, Carroll CL, Tang YS, et al. Factors associated with the development of severe asthma exacerbations in children. *J Asthma* 2011;48(6):558–64. https://doi.org/10.3109/02770903.2011.585411

10 Ungar WJ, Paterson JM, Gomes T, et al. Relationship of asthma management, socioeconomic status, and medication insurance characteristics to exacerbation frequency in children with asthma. *Ann Allergy Asthma Immunol* 2011;106(1):17–23. https://doi.org/10.1016/j.anai.2010.10.006

11 Stelmach I, Podlecka D, Smejda K, et al. Pediatric asthma caregiver's quality of life questionnaire is a useful tool for monitoring asthma in children. *Qual Life Res* 2012;21(9):1639–1642. https://doi.org/10.1007/s11136-011-0070-x

12 Milton B, Whitehead M, Holland P, et al. The social and economic consequences of childhood asthma across the lifecourse: a systematic review. *Child Care Health Dev* 2004;30(6):711–28. https://doi.org/10.1111/j.1365-2214.2004.00486.x

13 Szefler SJ, Phillips BR, Martinez FD, et al. Characterization of within-subject responses to fluticasone and montelukast in childhood asthma. *J Allergy Clin Immunol* 2005;115(2):233–42. https://doi.org/10.1016/j.jaci.2004.11.014

14 Lemanske RF, Mauger DT, Sorkness CA, et al. Step-up Therapy for Children with Uncontrolled Asthma While Receiving Inhaled Corticosteroids. *N Engl J Med* 2010;362(11):975–85. https://doi.org/10.1056/NEJMoa1001278

15 Chauhan BF, Chartrand C, Ni Chroinin M, et al. Addition of long-acting beta2-agonists to inhaled corticosteroids for chronic asthma in children. *Cochrane Database Syst Rev* 2015(11):CD007949. https://doi.org/10.1002/14651858.CD007949.pub2

16 Chauhan BF, Ben Salah R, Ducharme FM. Addition of anti-leukotriene agents to inhaled corticosteroids in children with persistent asthma. *Cochrane Database Syst Rev* 2013(10):CD009585. https://doi.org/10.1002/14651858.CD009585.pub2

For peer review only

17 Zhao Y, Han S, Shang J, et al. Effectiveness of drug treatment strategies to prevent asthma exacerbations and increase symptom-free days in asthmatic children: a network meta-analysis. *J Asthma* 2015;52(8):846–57. https://doi.org/10.3109/02770903.2015.1014101

18 van der Mark LB, Lyklema PH, Geskus RB, et al. A systematic review with attempted network meta-analysis of asthma therapy recommended for five to eighteen year olds in GINA steps three and four. *BMC Pulm Med* 2012;12:63. https://doi.org/10.1186/1471-2466-12-63

19 Hutton J, Williamson P. Bias in meta-analysis with variable selection within studies. *Applied Statistics* 2000;49:359–70. https://doi.org/10.1111/1467-9876.00197

20 Lenney W, McKay AJ, Tudur Smith C, et al. Management of Asthma in School age Children On Therapy (MASCOT): a randomised, double-blind, placebo-controlled, parallel study of efficacy and safety. *Health Technol Assess* 2013;17(4):1–218. https://doi.org/10.3310/hta17040

21 Lenney W, Perry S, Price D. Clinical trials and tribulations: the MASCOT study. *Thorax* 2011;66(6):457–8. https://doi.org/10.1136/thx.2010.156885

22 Moher D, Shamseer L, Clarke M, et al. Preferred reporting items for systematic review and meta-analysis protocols (PRISMA-P) 2015 statement. *Syst rev* 2015;4:1. https://doi.org/10.1186/2046-4053-4-1

23 Sinha IP, Gallagher R, Williamson PR, et al. Development of a core outcome set for clinical trials in childhood asthma: a survey of clinicians, parents, and young people. *Trials* 2012;13:103. https://doi.org/10.1186/1745-6215-13-103

24 Reddel HK, Taylor DR, Bateman ED, et al. An official American Thoracic Society/European Respiratory Society statement: asthma control and exacerbations: standardizing endpoints for clinical asthma trials and clinical practice. *Am J Respir Crit Care Med* 2009;180(1):59–99. https://doi.org/10.1164/rccm.200801-060ST

25 Nathan RA, Sorkness CA, Kosinski M, et al. Development of the asthma control test: a survey for assessing asthma control. *J Allergy Clin Immunol* 2004;113(1):59–65. https://doi.org/10.1016/j.jaci.2003.09.008

For peer review only

26 Juniper EF, O'Byrne PM, Guyatt GH, et al. Development and validation of a questionnaire to measure asthma control. *Eur Respir J* 1999;14:902–7. https://doi.org/10.1034/j.1399-3003.1999.14d29.x

27 Juniper EF, Svensson K, Mörk AC, et al. Measurement properties and interpretation of three shortened versions of the asthma control questionnaire. *Respir Med* 2005;99:553–8. https://doi.org/10.1016/j.rmed.2004.10.008

28 Juniper EF, Bousquet J, Abetz L, et al. Identifying 'well-controlled' and 'not well-controlled' asthma using the Asthma Control Questionnaire. *Respir Med* 2006;100:616–621. https://doi.org/10.1016/j.rmed.2005.08.012

29 Juniper EF, Gruffydd-Jones K, Ward S, et al. Asthma Control Questionnaire in Children: Validation, Measurement Properties, Interpretation. *Eur Respir J* 2010;36:1410–1416. https://doi.org/10.1183/09031936.00117509

30 Chauhan BF, Ducharme FM. Addition to inhaled corticosteroids of long-acting beta2-agonists versus anti-leukotrienes for chronic asthma. *Cochrane Database Syst Rev* 2014(1):CD003137. https://doi.org/10.1002/14651858.CD003137.pub5

31 Chauhan BF, Ducharme FM. Anti-leukotriene agents compared to inhaled corticosteroids in the management of recurrent and/or chronic asthma in adults and children. *Cochrane Database Syst Rev* 2012(5):CD002314. https://doi.org/10.1002/14651858.CD002314.pub3

32 Pruteanu AI, Chauhan BF, Zhang L, et al. Inhaled corticosteroids in children with persistent asthma: dose-response effects on growth. *Cochrane Database Syst Rev* 2014(7):CD009878. https://doi.org/10.1002/14651858.CD009878.pub2

33 Tudur Smith C, Hopkins C, Sydes MR, et al. How should individual participant data (IPD) from publicly funded clinical trials be shared? *BMC Med* 2015;13(1):1–7. https://doi.org/10.1186/s12916-015-0532-z

34 Higgins JPT, Altman DG, Gøtzsche PC, et al. The Cochrane Collaboration's tool for assessing risk of bias in randomised trials. *BMJ* 2011;343:d5928. https://doi.org/10.1136/bmj.d5928

For peer review only

35 The transitivity assumption CJC. Available from: https://airways.cochrane.org/sites/airways.cochrane.org/files/public/uploads/The transitivity assumption CJC.pdf

36 Dias S, Welton NJ, Caldwell DM, et al. Checking consistency in mixed treatment comparison meta-analysis. *Stat Med* 2010;29(7-8):932–44. https://doi.org/10.1002/sim.3767

37 Donegan S, Williamson P, D'Alessandro U, et al. Assessing key assumptions of network meta-analysis: a review of methods. *Res Synth Methods* 2013;4(4):291–323. https://doi.org/10.1002/jrsm.1085

38 Donegan S, Williamson P, D'Alessandro U, et al. Combining individual patient data and aggregate data in mixed treatment comparison meta-analysis: Individual patient data may be beneficial if only for a subset of trials. *Stat Med* 2013;32(6):914–30. https://doi.org/10.1002/sim.5584

39 Cooper NJ, Sutton AJ, Morris D, et al. Addressing between-study heterogeneity and inconsistency in mixed treatment comparisons: Application to stroke prevention treatments in individuals with non-rheumatic atrial fibrillation. *Stat Med* 2009;28(14):1861–81. https://doi.org/10.1002/sim.3594

40 Saramago P, Sutton AJ, Cooper NJ, et al. Mixed treatment comparisons using aggregate and individual participant level data. *Stat Med* 2012;31(28):3516–36. https://doi.org/10.1002/sim.5442

41 Donegan S, Williamson P, D'Alessandro U, et al. Assessing the consistency assumption by exploring treatment by covariate interactions in mixed treatment comparison meta-analysis: individual patient-level covariates versus aggregate trial-level covariates. *Stat Med* 2012;31(29):3840–57. https://doi.org/10.1002/sim.5470

42 Petrou S, Gray A. Economic evaluation using decision analytical modelling: design, conduct, analysis, and reporting. *BMJ* 2011;342:d1766. https://doi.org/10.1136/bmj.d1766

43 Husereau D, Drummond M, Petrou S, et al. Consolidated Health Economic Evaluation Reporting Standards (CHEERS) statement. *BMJ* 2013;346:f1049. https://doi.org/10.1136/bmj.f1049

For peer review only

44 National Institute for Health and Care Excellence. Guide to the methods of technology appraisal 2013. Available from: https://www.nice.org.uk/process/pmg9

45 NHS Improvement. NHS Reference costs 2017/18. https://improvement.nhs.uk/documents/6468/201718_reference_costs_data_and_guidance.zip

46 British National Formulary 79. March-September 2020

47 Ehteshami-Afshar S, Zafari Z, Hamidi N, et al. A Systematic Review of Decision-Analytic Models for Evaluating Cost-Effectiveness of Asthma Interventions. *Value Health* 2019;22(9):1070-1082. https://doi.org/10.1016/j.jval.2019.03.016

48 Dias S, Sutton AJ, Welton NJ, et al. Evidence synthesis for decision making 6: embedding evidence synthesis in probabilistic cost-effectiveness analysis. *Med Decis Making* 2013;33(5):671-8. https://doi.org/10.1177/0272989X13487257

49 Fenwick E, Claxton K, Sculpher M. Representing uncertainty: the role of cost-effectiveness acceptability curves. *Health Econ* 2001;10(8):779-87. https://doi.org/10.1002/hec.635

50 Claxton KP, Sculpher MJ. Using value of information analysis to prioritise health research: some lessons from recent UK experience. *Pharmacoeconomics* 2006;24(11):1055-68. https://doi.org/10.2165/00019053-200624110-00003

51 Strong M, Oakley JE, Brennan A. Estimating multi-parameter partial expected value of perfect information from a probabilistic sensitivity analysis sample: a non-parametric regression approach. *Med Decis Making* 2014;34(3):311-26. https://doi.org/10.1177/0272989X13505910

Figure caption

Figure 1 Treatment step-up for asthmatic patients according to age group. Adapted from GINA 2019.[1] SABA = Short-acting β_2-adrenoceptor agonist; ICS = Inhaled corticosteroid; LTRA = Leukotriene receptor antagonist; LABA = Long-acting β_2-adrenoceptor agonist; OCS = Oral corticosteroids ; IgE = Immunoglobulin E; IL = Interleukin

For peer review only

≤ 5-year-old (Children)

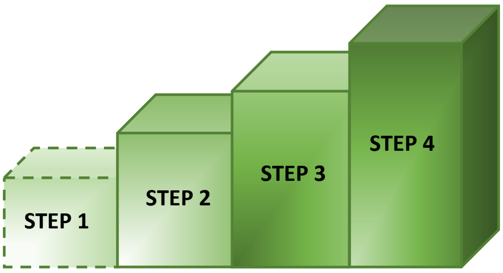

STEP 1 SABA as needed
STEP 2 Daily low dose ICS – SABA as needed.
Other options: LTRA, or intermittent ICS.
STEP 3 Double low dose ICS – SABA as needed
Other options: low dose ICS + LTRA.
STEP 4 Continue controller + specialist assessment
Other options: add LTRA / increase ICS frequency / add intermittent ICS.

6-11-year-old (Children)

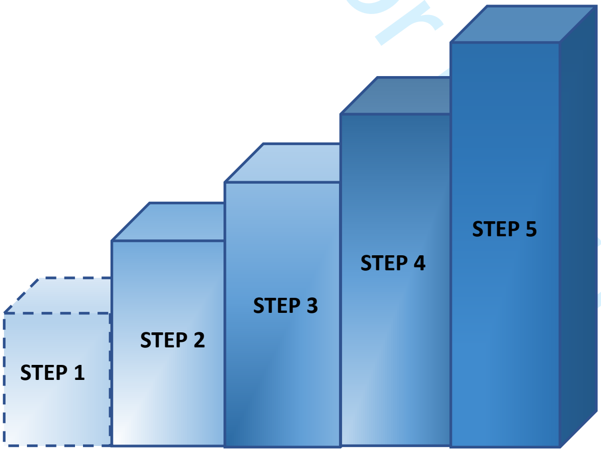

STEP 1 SABA as needed.
Other options: low dose of ICS taken with SABA, or daily low dose ICS.
STEP 2 Daily low dose ICS – SABA as needed.
Other options: LTRA, or low dose ICS taken with SABA.
STEP 3 Low dose ICS+LABA, or medium dose ICS – SABA as needed.
Other options: dose ICS+LTRA.
STEP 4 Medium dose ICS+LABA – SABA as needed.
Other options: High dose ICS+LABA, or add-on tiotropium, or add-on LTRA.
STEP 5 Phenotypic assessment ± add-on therapy (e.g., anti-IgE) – SABA as needed.
Other options: add-on anti-IL5, or add-on low dose OCS.

≥ 12-year-old (Adults and adolescents)

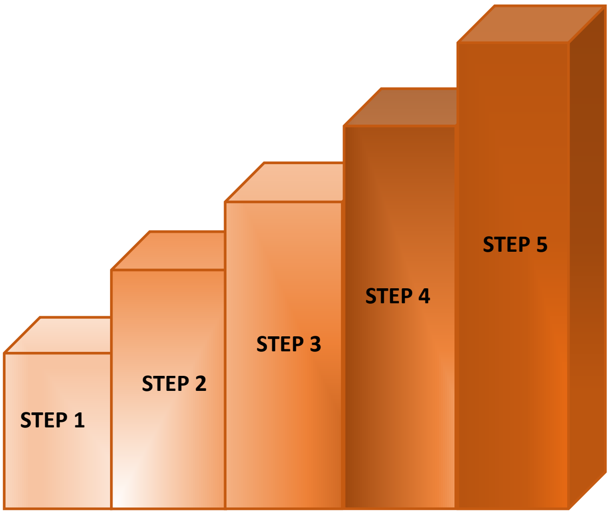

STEP 1 Low dose ICS/Formoterol as needed.
Other options: low dose of ICS taken with SABA.
STEP 2 Daily low dose ICS – Low dose ICS/Formoterol as needed.
Other options: LTRA, or low dose ICS taken with SABA.
STEP 3 Low dose ICS+LABA – Low dose ICS/Formoterol as needed.
Other options: Medium dose ICS, or low dose ICS+LTRA.
STEP 4 Medium dose ICS+LABA – Low dose ICS/Formoterol as needed.
Other options: High dose ICS, add-on tiotropium, or add-on LTRA.
STEP 5 High dose ICS+LABA. Phenotypic assessment ± add-on therapy (e.g., tiotropium, anti-IgE, anti-IL5/5R, anti-IL4R) – Low dose ICS/Formoterol as needed.
Other options: add-on low dose OCS.

Other reliever option for all steps: SABA as needed

For peer review only - http://bmjopen.bmj.com/site/about/guidelines.xhtml

EstablishINg the best STEp-up treatments for children with uncontrolled asthma despite INhaled corticosteroids (EINSTEIN): Protocol for a systematic review, network meta-analysis and cost effectiveness analysis using individual participant data (IPD)

Sofia Cividini[1], Ian Sinha[2], Sarah Donegan[1], Michelle Maden[3], Katie Rose[2], Olive Fulton[*], Dyfrig A. Hughes[4], Stephen Turner[5], Catrin Tudur Smith[1]

[1]Department of Biostatistics, University of Liverpool, Liverpool, UK
[2]Alder Hey Children's Foundation NHS Trust, Liverpool, UK
[3]Liverpool Reviews and Implementation Group (LRIG), University of Liverpool, Liverpool, UK
[4]Centre for Health Economics & Medicines Evaluation, Bangor University, Bangor, UK
[5]University Court of the University of Aberdeen, Aberdeen, UK
[*]Patients Representative, Liverpool, UK

For peer review only

Appendix 1. Example MEDLINE (OVID) search

1 exp Asthma/
2 asthma.ti,ab.
3 1 or 2
4 exp Infant/
5 infant*.ti,ab.
6 infancy.ti,ab.
7 newborn*.ti,ab.
8 baby*.ti,ab.
9 babies.ti,ab.
10 neonat*.ti,ab.
11 preterm*.ti,ab.
12 prematur*.ti,ab.
13 postmatur*.ti,ab.
14 exp child/
15 child*.ti,ab.
16 schoolchild*.ti,ab.
17 "school age*".ti,ab.
18 preschool*.ti,ab.
19 kid.ti,ab.
20 kids.ti,ab.
21 toddler*.ti,ab.
22 exp Adolescent/
23 adoles*.ti,ab.
24 teen*.ti,ab.
25 boy*.ti,ab.
26 girl*.ti,ab.
27 exp Minors/
28 minor*.ti,ab.
29 exp Puberty/
30 pubert*.ti,ab.
31 pubescen*.ti,ab.
32 prepubescen*.ti,ab.
33 exp Pediatrics/
34 paediatric*.ti,ab.
35 pediatric*.ti,ab.

36 exp Schools/
37 "nursery school*".ti,ab.
38 kindergar*.ti,ab.
39 "primary school*".ti,ab.
40 "secondary school*".ti,ab.
41 "elementary school*".ti,ab.
42 "high school*".ti,ab.
43 highschool*.ti,ab.
44 or/4-43
45 "inhaled corticosteroid*".mp.
46 ICS.mp.
47 exp Beclomethasone/
48 beclomethasone.mp.
49 "beclomethasone dipropionate".mp.
50 becotide.mp.
51 clenil.mp.
52 ciclesonide.mp.
53 "clenil modulite".mp.
54 exp Fluticasone/
55 "fluticasone propionate".mp.
56 fluticasone.mp.
57 flixotide.mp.
58 exp Budesonide/
59 budesonide.mp.
60 Mometasone Furoate/
61 mometasone.mp.
62 exp Adrenergic beta-Agonists/
63 "long acting beta-2 agonist*".mp.
64 "long acting beta2 agonist*".mp.
65 LABA.mp.
66 exp Formoterol Fumarate/
67 formoterol.mp.
68 Oxis.mp.
69 "fluticasone furoate".mp.
70 exp Salmeterol Xinafoate/
71 salmeterol.mp.
72 serevent.mp.
73 vilanterol.mp.
74 exp Leukotriene Antagonists/
75 "leukotriene receptor antagonist*".mp.
76 LTRA.mp.
77 zafirlukast.mp.
78 montelukast.mp.
79 exp Theophylline/
80 theophylline.mp.
81 Tiotropium.mp.
82 spiriva.mp.
83 Symbicort.mp.
84 Seretide.mp.
85 flutiform.mp.
86 relvar.mp.
87 or/45-86
88 Clinical Trial.pt.

For peer review only

89 Randomized Controlled Trial.pt.
90 exp Random Allocation/
91 exp Single-Blind Method/
92 exp Double-Blind Method/
93 exp Cross-Over Studies/
94 exp Placebos/
95 RCT.ti,ab.
96 Random*.ti,ab.
97 "Single blind*".ti,ab.
98 "Double blind*".ti,ab.
99 "triple blind*".ti,ab.
100 placebo*.ti,ab.
101 or/88-100
102 3 and 44 and 87 and 101
103 limit 102 to ed=20140701-20190911
104 limit 103 to english language
105 (case reports or editorial or letter).pt.
106 104 not 105

For peer review only

PRISMA-P 2015 Checklist

This checklist has been adapted for use with protocol submissions to *Systematic Reviews* from Table 3 in Moher D et al: Preferred reporting items for systematic review and meta-analysis protocols (PRISMA-P) 2015 statement. *Systematic Reviews* 2015 **4**:1

Section/topic	#	Checklist item	Information reported: Yes	Information reported: No	Line number(s)
**ADMINISTRATIVE INFORMATION**					
**Title**					
Identification	1a	Identify the report as a protocol of a systematic review	☑	☐	1-3
Update	1b	If the protocol is for an update of a previous systematic review, identify as such	☐	☐	
**Registration**	2	If registered, provide the name of the registry (e.g., PROSPERO) and registration number in the Abstract	☑	☐	65
**Authors**					
Contact	3a	Provide name, institutional affiliation, and e-mail address of all protocol authors; provide physical mailing address of corresponding author	☑	☐	6-21
Contributions	3b	Describe contributions of protocol authors and identify the guarantor of the review	☑	☐	445-451
**Amendments**	4	If the protocol represents an amendment of a previously completed or published protocol, identify as such and list changes; otherwise, state plan for documenting important protocol amendments	☐	☐	
**Support**					
Sources	5a	Indicate sources of financial or other support for the review	☑	☐	65-66<br>453-455
Sponsor	5b	Provide name for the review funder and/or sponsor	☑	☐	65-66<br>453-455
Role of sponsor/funder	5c	Describe roles of funder(s), sponsor(s), and/or institution(s), if any, in developing the protocol	☑	☐	453-455
**INTRODUCTION**					
**Rationale**	6	Describe the rationale for the review in the context of what is already known	☑	☐	81-162
**Objectives**	7	Provide an explicit statement of the question(s) the review will address with reference to participants, interventions, comparators, and outcomes (PICO)	☑	☐	163-183
**METHODS**					
**Eligibility criteria**	8	Specify the study characteristics (e.g., PICO, study design, setting, time frame) and report	☑	☐	187-254

For peer review only

BioMed Central
The Open Access Publisher

Section/topic	#	Checklist item	Information reported Yes	No	Line number(s)
		characteristics (e.g., years considered, language, publication status) to be used as criteria for eligibility for the review			
**Information sources**	9	Describe all intended information sources (e.g., electronic databases, contact with study authors, trial registers, or other grey literature sources) with planned dates of coverage	■	□	255-274
**Search strategy**	10	Present draft of search strategy to be used for at least one electronic database, including planned limits, such that it could be repeated	■	□	255-274
STUDY RECORDS					
Data management	11a	Describe the mechanism(s) that will be used to manage records and data throughout the review	■	□	276
Selection process	11b	State the process that will be used for selecting studies (e.g., two independent reviewers) through each phase of the review (i.e., screening, eligibility, and inclusion in meta-analysis)	■	□	279
Data collection process	11c	Describe planned method of extracting data from reports (e.g., piloting forms, done independently, in duplicate), any processes for obtaining and confirming data from investigators	■	□	285
**Data items**	12	List and define all variables for which data will be sought (e.g., PICO items, funding sources), any pre-planned data assumptions and simplifications	■	□	295
**Outcomes and prioritization**	13	List and define all outcomes for which data will be sought, including prioritization of main and additional outcomes, with rationale	■	□	309
**Risk of bias in individual studies**	14	Describe anticipated methods for assessing risk of bias of individual studies, including whether this will be done at the outcome or study level, or both; state how this information will be used in data synthesis	■	□	345
DATA					
**Synthesis**	15a	Describe criteria under which study data will be quantitatively synthesized	■	□	350
	15b	If data are appropriate for quantitative synthesis, describe planned summary measures, methods of handling data, and methods of combining data from studies, including any planned exploration of consistency (e.g., I^2, Kendall's tau)	■	□	
	15c	Describe any proposed additional analyses (e.g., sensitivity or subgroup analyses, meta-regression)	■	□	
	15d	If quantitative synthesis is not appropriate, describe the type of summary planned	■	□	
**Meta-bias(es)**	16	Specify any planned assessment of meta-bias(es) (e.g., publication bias across studies, selective reporting within studies)	■	□	396
**Confidence in cumulative evidence**	17	Describe how the strength of the body of evidence will be assessed (e.g., GRADE)	■	□	402

For peer review only

BioMed Central The Open Access Publisher

BMJ Open

EstablishINg the best STEp-up treatments for children with uncontrolled asthma despite INhaled corticosteroids (EINSTEIN): Protocol for a systematic review, network meta-analysis and cost-effectiveness analysis using individual participant data (IPD)

Journal:	*BMJ Open*
Manuscript ID	bmjopen-2020-040528.R1
Article Type:	Protocol
Date Submitted by the Author:	16-Nov-2020
Complete List of Authors:	Cividini, Sofia; University of Liverpool, Biostatistics<br>Sinha, Ian; Alder Hey Children's NHS Foundation Trust<br>Donegan, Sarah; University of Liverpool, Biostatistics<br>Maden, Michelle; University of Liverpool, Liverpool Reviews and Implementation Group<br>Culeddu, Giovanna; Bangor University, Centre for Health Economics and Medicines Evaluation<br>Rose, Katie; Alder Hey Children's NHS Foundation Trust<br>Fulton, Olive; Patients Representative<br>Hughes, Dyfrig; Bangor University, Centre for Health Economics and Medicines Evaluation<br>Turner, Stephen; University Court of the University of Aberdeen, Department of Child Health<br>Tudur-Smith, Catrin; University of Liverpool, Biostatistics
**Primary Subject Heading**:	Respiratory medicine
Secondary Subject Heading:	Epidemiology, Evidence based practice, Health economics, Paediatrics, Research methods
Keywords:	STATISTICS & RESEARCH METHODS, Asthma < THORACIC MEDICINE, Epidemiology < THORACIC MEDICINE, PAEDIATRICS

SCHOLARONE™
Manuscripts

I, the Submitting Author has the right to grant and does grant on behalf of all authors of the Work (as defined in the below author licence), an exclusive licence and/or a non-exclusive licence for contributions from authors who are: i) UK Crown employees; ii) where BMJ has agreed a CC-BY licence shall apply, and/or iii) in accordance with the terms applicable for US Federal Government officers or employees acting as part of their official duties; on a worldwide, perpetual, irrevocable, royalty-free basis to BMJ Publishing Group Ltd ("BMJ") its licensees and where the relevant Journal is co-owned by BMJ to the co-owners of the Journal, to publish the Work in this journal and any other BMJ products and to exploit all rights, as set out in our licence.

The Submitting Author accepts and understands that any supply made under these terms is made by BMJ to the Submitting Author unless you are acting as an employee on behalf of your employer or a postgraduate student of an affiliated institution which is paying any applicable article publishing charge ("APC") for Open Access articles. Where the Submitting Author wishes to make the Work available on an Open Access basis (and intends to pay the relevant APC), the terms of reuse of such Open Access shall be governed by a Creative Commons licence – details of these licences and which Creative Commons licence will apply to this Work are set out in our licence referred to above.

Other than as permitted in any relevant BMJ Author's Self Archiving Policies, I confirm this Work has not been accepted for publication elsewhere, is not being considered for publication elsewhere and does not duplicate material already published. I confirm all authors consent to publication of this Work and authorise the granting of this licence.

For peer review only

EstablishINg the best STEp-up treatments for children with uncontrolled asthma despite INhaled corticosteroids (EINSTEIN): Protocol for a systematic review, network meta-analysis and cost-effectiveness analysis using individual participant data (IPD)

Sofia Cividini[1], Ian Sinha[2§], Sarah Donegan[1], Michelle Maden[3], Giovanna Culeddu[4], Katie Rose[2], Olive Fulton[*], Dyfrig A. Hughes[4], Stephen Turner[5], Catrin Tudur Smith[1§**]

[1]Department of Biostatistics, University of Liverpool, Liverpool, UK
Catrin Tudur Smith – Institute of Translational Medicine, Department of Biostatistics, University of Liverpool, Crown Street, Liverpool L693BX, UK – cat1@liverpool.ac.uk – Tel. +44 (0)151 794 4059/+44 (0)151 794 9754 – ORCID: https://orcid.org/0000-0003-3051-1445
Sofia Cividini – Sofia.Cividini@liverpool.ac.uk – ORCID: https://orcid.org/0000-0003-2705-9224
Sarah Donegan – Sarah.Donegan@liverpool.ac.uk – ORCID: https://orcid.org/0000-0003-1709-2290
[2]Alder Hey Children's Foundation NHS Trust, Liverpool, UK
Ian Sinha – Ian.Sinha@alderhey.nhs.uk – ORCID: https://orcid.org/0000-0002-7342-5523
Katie Rose – katierose100@gmail.com – ORCID: https://orcid.org/0000-0002-2348-2036
[3]Liverpool Reviews and Implementation Group (LRIG), University of Liverpool, Liverpool, UK
Michelle Maden – mmaden@liverpool.ac.uk – ORCID: https://orcid.org/0000-0003-4419-6343
[4]Centre for Health Economics & Medicines Evaluation, Bangor University, Bangor, UK
Dyfrig Hughes – d.a.hughes@bangor.ac.uk – ORCID: https://orcid.org/0000-0001-8247-7459
Giovanna Culeddu – g.culeddu@bangor.ac.uk – ORCID: https://orcid.org/0000-0001-5032-4255
[5]University Court of the University of Aberdeen, Aberdeen, UK
Stephen Turner – s.w.turner@abdn.ac.uk – ORCID: https://orcid.org/0000-0001-8393-5060
[*]Patients Representative, Liverpool, UK
Olive Fulton – olive.fulton@gmail.com

Running head: The EINSTEIN Study Protocol (IPD-NMA)

[**]Correspondence to Professor Catrin Tudur Smith; cat1@liverpool.ac.uk

[§]CTS and IS are joint senior authors

ABSTRACT

Introduction

Asthma affects millions of children worldwide – 1.1 million children in the UK. Asthma symptoms cannot be cured but can be palliated with low dose inhaled corticosteroids (ICS) in the majority of individuals. Treatment with a low dose ICS, however, fails to control asthma symptoms in around 10-15% of children and this places the individual at increased risk for an asthma attack. At present, there is no clear preferred treatment option for a child whose asthma is not controlled by low dose ICS, and international guidelines currently recommend at least three treatment options. Herein we propose a systematic review and individual participant data network meta-analysis (IPD-NMA)

For peer review only

aiming to synthesise all available published and unpublished evidence from randomised controlled trials (RCTs) to establish the clinical effectiveness of pharmacological treatments in children and adolescents with uncontrolled asthma on ICS and help to make evidence-informed treatment choices. This will be used to parameterise a Markov-based economic model to assess the cost-effectiveness of alternative treatment options in order to inform decisions in the context of drug formularies and clinical guidelines.

Methods and analysis

We will search in MEDLINE, the Cochrane Library, the Cochrane Central Register of Controlled Trials (CENTRAL), Embase, NICE Technology Appraisals, and the NIHR HTA series for RCTs of interventions in patients with uncontrolled asthma on ICS. All studies where children and adolescents were eligible for inclusion will be considered, and authors or sponsors will be contacted to request IPD on patients aged <18. The reference lists of existing clinical guidelines, along with included studies and relevant reviews, will be checked to identify further relevant studies. Unpublished studies will be located by searching across a range of clinical trial registries, including internal trial registers for pharmaceutical companies. All studies will be appraised for inclusion against predefined inclusion and exclusion criteria by two independent reviewers with disagreements resolved through discussion with a third reviewer. We will perform an IPD-NMA – eventually supplemented with aggregate data for the RCTs without IPD – to establish both the probability that a treatment is best and the probability that a particular treatment is most likely to be effective for a specific profile of the patient. The IPD-NMA will be performed for each outcome variable within a Bayesian framework, using the WinBUGS software. Also, potential patient-level characteristics that may modify treatment effects will be explored, which represents one of the strengths of this study.

Ethics and dissemination

The University of Liverpool committee on Research Ethics has confirmed that ethics review is not required. The dissemination plan consists of publishing the results in an open-access medical

For peer review only

journal, a plain-language summary available for parents and children, dissemination via local, national, and international meetings and conferences, and the Press Offices of our HEIs. A synopsis of results will be disseminated to NICE and BTS/SIGN as highly relevant to future clinical guideline updates.

PROSPERO registration number: CRD42019127599 – This project is funded by the NIHR Health Technology Assessment Programme (project number 16/110/16).

Keywords inhaled corticosteroids ▪ long-acting β_2-agonists ▪ leukotriene receptor antagonist ▪ theophylline ▪ exacerbation ▪ asthma control ▪ hospital admissions ▪ adolescents

For peer review only

Strengths and limitations of this study

- This study will be the most comprehensive synthesis of interventional studies of children and adolescents with uncontrolled asthma on ICS.
- It will be able to establish the most clinically and cost-effective step-up therapy for asthma, also considering those treatments where no head-to-head comparison exists, providing evidence to make informed treatment choices.
- It will be able to evaluate the influence of patient-level characteristics on treatment effect.
- It will provide an assessment of the incremental cost per quality-adjusted life-year gained of treatment options, from the perspective of the NHS and personal social services.
- Individual participant data may not be available from all eligible RCTs and integration with aggregate data may be needed.

INTRODUCTION

Rationale

Recent epidemiological data show that an always-increasing number of people of all age group worldwide, among that millions of children and adolescents, are affected by asthma with a notable social and economic burden for health systems.[1] Asthma remains a common medical condition affecting over one million children in the UK, one of the highest prevalence rate worldwide.[2, 3]

Asthma is characterised by symptoms of wheeze, breathlessness, chest-tightness, cough and can affect the child's quality of life by limiting daily activities and causing acute attacks. In the UK, it was estimated that one child is admitted to the hospital every 20 minutes because of an asthma attack.[2]

The British Guideline[4, 5] on the management of asthma recommends that, following a diagnosis of asthma in a child, a stepwise approach to treatment should be taken. The initial level of treatment is a low dose inhaled corticosteroid to prevent symptoms plus a short acting β_2-adrenoceptor agonist (SABA) to relieve symptoms. However, treatment with a low dose ICS fails to control asthma symptoms in around 10-15% of children.[6] If asthma remains uncontrolled, a series of further steps are followed consisting of a treatment step-up by including add-on preventer therapies such as long-acting β_2-adrenoceptor agonists (LABA) or leukotriene receptor antagonists (LTRA), increasing the dose of ICS, or adding sustained-release (SR) theophylline.[1, 4, 5]

Choosing the best step-up treatment thus becomes a crucial decision to prevent occurring of exacerbations and to avoid poor asthma control, also improving the quality of life of patients and their families and optimising the use of NHS resources.[7-12] At present, no clear preferential option for initial step-up exists, and the decision-making process about treatment choices mainly bases on clinicians' preferences and not on evidence-based practice. Moreover, there is substantial heterogeneity among individuals in the treatment response.[13, 14]

A recent Cochrane review[15] comprising of 33 trials with 6,381 children demonstrated that adding LABAs to ICSs was not associated with a significant decrease in the rate of exacerbations requiring systemic steroids but was superior for improving lung function (e.g., FEV_1 and PEF), compared with the same or higher doses of ICS. No statistically significant group difference in hospital admissions was found. Notwithstanding, because of a trend towards increased risk of hospital admission with LABA independently of ICS dose, the authors advised further monitoring of treatment.[15]

For peer review only

Another Cochrane review[16] published in 2013 found that adding LABAs to ICSs was associated with no statistically significant reduction neither of the use of oral corticosteroids as rescue nor hospital admissions compared to the same or an increased dose of ICS. Evidence was based only on 4 trials with 559 children and adolescents having mild to moderate asthma.[16] The authors cautioned that the lack of paediatric trials, the absence of data on pre-schoolers, and the variability in the reporting of relevant clinical outcomes was a limit for firm conclusions regarding this comparison.[16]

Two network meta-analyses (NMAs), analysing published RCT-level aggregate data for children with uncontrolled asthma, have already been conducted,[17, 18] but the evidence from these analyses has important limitations. The considerable variation in, and the incomplete reporting of outcome measurements across RCTs prevented a formal NMA and assessment of relative efficacies of treatments in one NMA of 23 RCTs (4,129 patients).[18] In 2015, Zhao et al.[17] conducted a formal NMA consisting of 35 RCTs with 12,010 children. These authors suggested that combined ICS and LABA treatments were most effective in preventing exacerbations and that treatments with medium- or high-dose ICS, combined ICS/LTRA, and low-dose ICS seem to be equally effective.[17] However, the analysis has limitations as the authors excluded 70 relevant RCTs because publications of these RCTs did not provide data about 'exacerbations' or 'symptom-free days'. Outcome reporting bias[19] is, therefore, a severe threat to the validity of their results if the excluded studies had selectively reported results based on the statistical significance of their findings. Moreover, a series of other issues make the interpretation and generalisability of the results of this NMA difficult. There is a lack of complete overlapping of the included studies compared to the previous Cochrane reviews[15, 16] and previous planned NMA[18] – only 7 RCTs are in common across the two NMAs.[17, 18] Two other weak points are represented by the lack of analyses focussing on outcomes of importance and relevance to patients and the lack of analyses comparing different drugs (ICS, LABA, LTRA), doses, and types of inhalation devices within ICS

For peer review only

and LABA classes. We have also included tiotropium in the search algorithm as a long-acting antimuscarinic medication (LAMA), but we are not confident of finding sufficient studies.

Several randomised clinical trials (RCTs) have compared two alternative step-up options head-to-head, but only a few individual trials have analysed more than two classes head-to-head. The BADGER trial[14] randomised 182 children and adolescents with uncontrolled asthma on fluticasone to receive each of the following therapies: 'ICS step-up', 'LABA step-up', and 'LTRA step-up', obtaining differential responses to every step-up treatment. 'LABA step-up' showed significantly more likely to provide the 'best' response; yet, many patients better replied with 'ICS or LTRA step-up' suggesting that for some patients, these treatments may be preferred.[14] Overall, results from BADGER trial were heterogeneous, and a 16-week follow-up may not be sufficient.

The MASCOT trial[20] randomised children aged 6-14 to fluticasone plus placebo, fluticasone/salmeterol plus placebo, and fluticasone plus montelukast. However, this RCT failed to recruit an adequate number of patients due to significant organisational challenges, issues of accessing patients from primary care, and changes in prescribing habits during the trial. These challenges limit the potential success of similar future clinical trials, and recommendations have been made for the use of alternative study designs[20, 21] to compare alternative step-up treatments.

There is thus an urgent need for a novel approach to be taken to synthesise all of the available evidence using robust, unbiased methods, also including unpublished data. A key strength of the EINSTEIN analysis will be the assessment of individual patient-level characteristics in modifying the patient's response to treatment. No study has previously considered this aspect, although it represents a crucial clinical step in evaluating treatment efficacy. Furthermore, an economic analysis of the treatment options will provide evidence on their cost-effectiveness from the perspective of the National Health Service in the UK. Results from the EINSTEIN study will provide clinicians and patients with accessible, high-quality, patient-relevant information to help make evidence-informed treatment choices. Earlier identification of the best step-up treatment for a

For peer review only

particular child could have a significant impact on children's lives with more extensive benefits to society and the NHS.

Objectives

Our research objective is to establish the best treatment option for patients younger than 18 whose asthma symptoms are uncontrolled by low-medium dose ICS. To this goal, the EINSTEIN study will identify and synthesise all available evidence from RCTs to establish the effectiveness of pharmacological treatments in children and adolescents aged <18 having poor asthma control on ICS. The study will also investigate the modifiers of treatment effect (e.g., age, ethnicity, asthmatic phenotype) to optimise a targeted therapy and maximise patients' informed choice of treatment. The economic analysis will estimate the cost-effectiveness of treatments in terms of their incremental costs per quality-adjusted life-year gained.

Specifically, we aim to:

1. Conduct a systematic review to identify relevant RCTs of treatment with children and adolescents whose asthma remains uncontrolled despite ICS use.
2. Collect individual participant data (IPD) from all eligible trials.
3. Carry out a network meta-analysis of IPD to identify the most effective treatment.
4. Identify modifiers of treatment effect to establish which patients respond better to each treatment.
5. Develop an economic model to estimate the incremental cost per quality-adjusted life-year gained of treatment options, from the perspective of the NHS and Personal Social Services.
6. Identify remaining uncertainties formulating recommendations to inform priorities aimed at future research.
7. Disseminate findings.

METHODS

For peer review only

The PRISMA-P guidelines (Preferred Reporting Items for Systematic Reviews and Meta-Analysis Protocols)[22] have guided the drafting of this protocol.

Eligibility criteria

For inclusion in the EINSTEIN study, we will select studies according to the criteria outlined below.

Study designs

We will include parallel and cross-over randomised controlled trials (RCTs), of any duration, using any level of blinding and comparing at least one of the health technologies of interest. All RCTs meeting our inclusion criteria will be included irrespective of the outcomes reported in the publication, to reduce the potential for outcome reporting bias.

Participants

We will focus on children (<12 years) and adolescents (12-17 years) of any ethnicity with poor asthma control on ICS before randomisation as defined by the study protocol. We will include studies with mixed age groups in which children and adolescents were eligible because we will contact authors concerning specific data on patients aged <18. Patients aged ≥18, or with stable asthma as defined by the study authors, or using an asthma medication other than ICS, or an ICS with an add-on active (e.g., ICS/LABA) as treatment regimen before randomisation will not be considered. Participants using any dose ICS at randomisation will be eligible for inclusion.

Interventions

We will consider as eligible all studies where children and adolescents were randomised to at least one of the following treatments:

- Inhaled Corticosteroids (ICS), alone or in combination with other treatments – beclometasone dipropionate; ciclesonide; fluticasone propionate; fluticasone furoate; budesonide; mometasone.
- Long-acting β_2-agonists (LABA) – formoterol; salmeterol; vilanterol.
- Leukotriene receptor antagonists (LTRA) – zafirlukast; montelukast.

For peer review only

▪ Theophylline.

Any dose of preventer treatment – oral or inhaled, and any inhaler devices (pressurised metered-dose inhaler, dry powder inhaler, combination inhaler) – will be considered. Presence or absence of spacer devices will be noted.

We will not consider studies with participants randomised to any asthma medication other than those listed in the inclusion criteria

Comparators

For both primary and secondary outcomes – if not specified otherwise – patient outcomes will be compared at two levels, namely among:

1. Every class of treatment – ICS (alone or with add-on actives), LABA, LTRA, theophylline, and placebo. For ICS, we will distinguish between low, medium, and high doses as defined in GINA – Global Initiative for Asthma 2019[1] (Table 1 and Figure 1).

Table 1 Estimated clinical comparability daily doses (µg) of Inhaled Corticosteroids (ICS)

**≤ 5-year-old (Children)**			
**Drug**	**Low Dose**	**Medium Dose**	**High Dose**
Beclomethasone dipropionate (HFA)	100 (≥5 years)	N.A.	N.A.
Budesonide nebulised	500 (≥1 year)	N.A.	N.A.
Budesonide pMDI + spacer	N.A.	N.A.	N.A.
Fluticasone propionate (HFA)	50 (≥4 years)	N.A.	N.A.
Mometasone furoate	110 (≥4 years)	N.A.	N.A.
Ciclesonide	N.A.	N.A.	N.A.
**6-11-year-old (Children)**			
**Drug**	**Low Dose**	**Medium Dose**	**High Dose**
Beclomethasone dipropionate (CFC)	100-200	>200-400	>400
Beclomethasone dipropionate (HFA)	50-100	>100-200	>200
Budesonide (DPI)	100-200	>200-400	>400
Budesonide (nebules)	250-500	>500-1000	>1000
Ciclesonide	80	>80-160	>160
Fluticasone furoate (DPI)	N.A.	N.A.	N.A.
Fluticasone propionate (DPI)	100-200	>200-400	>400
Fluticasone propionate (HFA)	100-200	>200-500	>500
Mometasone furoate	110	≥220-<440	≥440
**≥ 12-year-old (Adults and adolescents)**			
**Drug**	**Low Dose**	**Medium Dose**	**High Dose**
Beclomethasone dipropionate (CFC)	200-500	>500-1000	>1000
Beclomethasone dipropionate (HFA)	100-200	>200-400	>400
Budesonide (DPI)	200-400	>400-800	>800
Ciclesonide (HFA)	80-160	>160-320	>320

For peer review only

Fluticasone furoate (DPI)	100	N.A.	200
Fluticasone propionate (DPI)	100-250	>250-500	>500
Fluticasone propionate (HFA)	100-250	>250-500	>500
Mometasone furoate	110-220	>220-440	>440

CFC = chlorofluorocarbon propellant (no longer used; included for comparison with older literature)
DPI = dry powder inhaler
HFA = hydrofluoroalkane propellant
N.A. = not applicable

2. Every drugs within every class of treatment – beclometasone dipropionate; ciclesonide; fluticasone propionate; fluticasone furoate; budesonide; mometasone; formoterol; salmeterol; vilanterol; zafirlukast; montelukast; theophylline; (placebo).

Moreover, we will explore complex hierarchical models that consider both classes of treatments and actives with interactions of covariates. Healthy controls will not be considered as an appropriate reference group.

Outcomes

Review outcomes will include the outcomes identified as important to healthcare practitioners and patients during the development of a core outcome set for trials in children with asthma.[23, 24]

Primary outcomes:

- Exacerbations as defined by ERS/ATS.[24]
- Asthma control measured by a validated test (e.g., ACT,[25] ACQ,[26-29] other).

Secondary outcomes:

- Symptoms/symptom score
- Quality of Life (QoL)
- Mortality – although rare, this is an important outcome for parents
- Physiological outcomes (e.g., FEV_1, bronchial responsiveness)
- Adverse effects (AEs) – including growth and withdrawals due to adverse effects
- Hospital admissions
- Total health care costs (obtained by the sum-product of item of resource use and their unit cost), resource use, and utility outcomes to inform the economic model.

For peer review only

Timing

Studies will be selected for inclusion independently of the length of follow-up of outcomes.

Setting

There will be no restrictions by type of setting.

Language

We will include only articles reported in the English language.

Information sources and search strategy

Our search strategy will identify published and unpublished studies and builds upon previously published search strategies for aggregate data network meta-analyses[17, 18] and Cochrane reviews.[15, 16, 30-32] (see Supplementary File 1; for example, MEDLINE search). The search strategies will run from 2014 onwards only. From inception to 2013, we will consider as exhaustive the searches already carried out for the meta-analyses and Cochrane reviews above mentioned,[15-18, 30-32] and we will retrieve all the corresponding studies.

We will search MEDLINE, the Cochrane Library, the Cochrane Central Register of Controlled Trials (CENTRAL), Embase, NICE Technology Appraisals, and the NIHR HTA series using relevant search terms. We will also scan the reference list of included studies and relevant reviews along with the reference lists of existing clinical guidelines such as the British Thoracic Society (BTS) Guideline[4, 5] and GINA.[1]

For unpublished studies, we will search a range of clinical trial registries enclosed inside the WHO 'International Clinical Trials Registry Platform' search portal (including clinicaltrials.gov and ISRCTN) and conference abstracts (e.g., European Respiratory Society; American Thoracic Society).

We will also search internal clinical trial registers for pharmaceutical companies that manufacture health technologies of interest (e.g., GSK, AstraZeneca, Novartis, Merck).

For peer review only

We will limit the search to English language articles, and we will verify that RCTs have included participants aged <18 as a subset.

STUDY RECORDS

Data management

Studies identified for screening of abstracts and full-text articles will be managed within Covidence (https://www.covidence.org/home), a systematic review management tool.

Selection process

All studies will be screened and assessed for inclusion by two independent reviewers (SC and KR) with disagreements resolved through discussion with a third reviewer (IS). To minimise the impact of selective outcome reporting, we will include all RCTs meeting our inclusion criteria irrespectively of the outcome(s) reported in the publication. The authors will thus be contacted to ask for information and the IPD for all outcomes relevant to the review.

Data collection process

The first author, or sponsor, of each included trial will be approached and asked to supply anonymised IPD, metadata, and relevant documentation[33] (protocol and blank case report forms) from the respective trial, either through direct contact, or through data sharing portals such as clinicalstudydatarequest.com, where relevant.

Before starting the quantitative synthesis, we will conduct a set of standard quality and consistency checks on the provided data. This will involve cross-checks by re-analysing IPD against previously published results to highlight inconsistencies or possible errors. Any queries will be raised with the original trialists wherever possible. Data will be cleaned and standardised to allow pooling and subsequent analyses of the data.

Data items

The data requested from each clinical trial will include at least:

For peer review only

- Baseline characteristics – age; sex; ethnicity; eczema; height; weight; baseline severity; baseline Peak Expiratory Flow Rate (PEFR); Forced Expiratory Volume in one second (FEV_1). Where available, we will also collect other variables such as age at asthma diagnosis, asthma-related hospitalization and exacerbation history, allergic sensitisation, parental asthma, objective indicators of asthma such as bronchodilator reversibility or methacholine PC20.
- The date of randomisation and dates of follow-up visits/interval between randomisation and follow up.
- Treatment details including inhalation device and dose.
- Adherence data if available.
- Data for the review outcomes along with details of their definitions and measurement tools used – symptoms; exacerbations; asthma control; mortality; quality of life; growth; physiological outcomes: Peak Expiratory Flow Rate (PEFR) and Forced Expiratory Volume in one second (FEV_1); hospital admissions.
- Cost, resource use, and utility outcomes to inform the economic model.

We will use data from the intention-to-treat (ITT) patients.

Outcomes and prioritisation

Primary outcomes

The primary outcomes of the study are exacerbations and loss of/change in asthma control measured by a validated test.

1. Exacerbations are defined as in ERS/ATS,[24] namely "*events characterized by a change from the patient's previous status*". We will define exacerbations as every event requiring the use of oral corticosteroids (OCS), the need for unscheduled visits with general practitioners (GPs), or at the emergency department (ED), and hospitalisation.
2. Based on the definition reported in ERS/ATS,[24] asthma control corresponds to "*the extent to which the various manifestations of asthma have been reduced or removed by treatment*".

For peer review only

Exacerbations are expected to be an individual-level numerical variable (binary, ordinal, or count) or an individual-level time-to-event variable. Asthma control is expected to be an individual-level numerical variable (categorical) and will be measured by a validated test (e.g., ACT,[25] ACQ,[26-29], other). Table 2 represents a possible categorisation for asthma control outcome based on ACT/ACQ scores. A standardisation method coherently to ACT and ACQ will be applied for other kinds of test. After consultation with clinicians, we will decide if, for this outcome variable, it is worthier to consider the full longitudinal profile (multiple measurements) or to establish a clinically relevant time-point.

Table 2 A possible categorisation of asthma control outcome based on ACT/ACQ scores

Test	Asthma control	Total score
**ACT 4-11 (years)**	0 = poor control	score ≤19
	1 = good/total control	score = 20–27
**ACT 12+ (years)**	0 = poor control	score ≤19
	1 = good/total control	score = 20–25
**ACQ**	0 = poor control	score >1
	1 = good/total control	score ≤1
**Others**	0 = poor control	to be evaluated on an individual case by case basis
	1 = good/total control	

Note: if both ACT and ACQ are present, we will choose ACT as the best choice.

Secondary outcomes

The secondary outcomes of the study are as follows.

1. Symptoms/symptom score – The mode to report symptoms or symptom score may be different across studies. We will distinguish between daytime and night-time symptoms, whatever they are, also considering their intensity.
2. Quality of life – QoL may have been measured differently across the studies. We will use the most frequent scale as a model to transform the remaining data using, e.g., established conversion tables.
3. Mortality – This variable may be reported in different ways across studies. It will always be converted to a binary variable.

For peer review only

4. Physiological measurements – FEV_1 in L/s and PEFR%. After consulting clinicians, we will decide if considering the full longitudinal profile (multiple measures) or establishing a clinically relevant time-point.
5. Adverse events – These may differ across the studies. We will consider relevant AEs, such as cardiac and neuropsychiatric disorders, infections, pneumonia, behavioural difficulty, growth, and diabetes.
6. Hospital admissions – This variable may be reported in different ways across studies. We will look for evidence such as dates of hospitalisation, adverse events indicating admission occurred, and variables directly reporting hospitalisation, between the randomisation visit and the end of the follow-up.

Economic outcomes

7. The primary economic outcome will be the incremental cost per quality-adjusted life-year (QALY) gained. This will be estimated from data on:
    a. Hospital admissions
    b. Other items resource use, costs, and utilities – These will be based on purposive searches of the economic and medical literature.

Risk of bias in individual studies

Two independent reviewers will use the Cochrane Risk of Bias tool[34] to record risk of bias concerning randomisation method, allocation concealment, blinding, incomplete outcome data, and selective reporting. Disagreements will be resolved through discussion with a third reviewer.

DATA

Synthesis

For every separate outcome, we will create a NMA diagram displaying the number of studies and patients for each treatment comparison within the network.

For peer review only

We will fit a Bayesian hierarchical meta-analysis model – supplemented with aggregate data if necessary – to synthesise the available IPD. We will thus estimate the relative treatment effect – odds ratio (OR) for categorical data and difference in means for continuous data – and credibility interval for every pair-wise comparison based on direct evidence. We will assess the homogeneity assumption by comparing the Device Information Criterion (DIC) of fixed-effect and random-effect models and observing the between trial variance. The forest plots, chi-square test for heterogeneity, and I^2 statistic will be examined to assess the evidence of heterogeneity within each pair-wise meta-analysis based on direct evidence.

We will conduct a NMA for every outcome within a Bayesian framework with the WinBUGS software with the Goodness-of-fit assessed by calculating the posterior mean residual deviance with DIC used as a basis for model comparison. Correlation between treatment effects from multi-arm trials will be appropriately accounted for. From the NMA, we can estimate the relative treatment effect for every pair-wise comparison irrespective of they have been compared directly in an RCT as well as calculating the probability that each treatment is the best, or worst, for a given outcome. In random-effects NMA models, we will check the conventional assumption that between-trial variance is the same for each comparison, by fitting pair-wise models based on direct evidence and assessing whether the variance is similar for each comparison. If the assumption appears unrealistic, we will explore other variance structures for the NMA model.

In a NMA, the indirect comparisons are not protected by randomisation, as for direct comparisons. So, they may be affected by confounding due to differences among clinical trials.[35] The validity of a NMA thus depends on the critical assumption of transitivity namely, to verify whether the probability of having been able to have given any of the treatment in the network to any patient in the network was equally likely.[35] We will check this assumption by comparing the inclusion/exclusion criteria of trials to make a judgement about whether patients, trial protocols, doses, administration procedures, and so on are similar in the modes that might modify treatment

For peer review only

effect. We will use model fit and selection statistics to informally assess whether inconsistency is evident along with a formal analysis using a 'node-splitting' approach.[36, 37]

We will explore potential patient-level characteristics that may modify treatment effects using hierarchical models with interaction effects between treatment and specific covariates. Initially, based on direct evidence, and subsequently in a NMA of the IPD, with aggregate data if IPD is unavailable.[38-41] The effect of covariates will be separated within and between trials. The underlying consistency assumption of these models will also be explored. Patient-level characteristics of interest include age, gender, ethnicity, eczema status, asthmatic phenotype, baseline %PEFR, and asthma severity. A literature search will be conducted to identify any further potential characteristics to explore.

We will fit a NMA model to compare compounds within each class and separate models to compare the different classes of treatments. Furthermore, complex hierarchical models that account for both classes and compounds with covariate interactions will be explored.

We will undertake sensitivity analyses for investigating the impact of missing IPD at the level of results and conclusions of NMA and for exploring the robustness of results to different priors in the Bayesian analyses. If sufficient data are available, sensitivity analyses that adjust for levels of adherence will be conducted to assess the robustness of results obtained from the primary analysis on the ITT patients.

Meta-bias(es)

To minimise the impact of selective outcome reporting, we will include all RCTs meeting our inclusion criteria irrespectively of the outcome(s) reported in the publication. For analyses containing 10 or more trials – due to the low power of the assessments for analyses containing small numbers of trials – we will investigate publication bias (and other selection bias/small study effects) by using funnel plots and appropriate statistical tests for funnel plot asymmetry.

Confidence in cumulative evidence

For peer review only

419 We will assess the quality of evidence for all outcomes across the domains of risk of bias, consistency, directness, precision, and publication bias according to GRADE (Grading of Recommendations Assessment, Development and Evaluation).

422 **Economic analysis**

423 The economic analysis will be conducted using standard methods,[42] based on the most robust data available and reported according to the CHEERS statement.[43] Cost-effectiveness will be estimated from the perspective of the NHS and Personal Social Services in line with NICE guidance[44] and based on an economic model that considers health outcomes, resource use, costs, and health utilities to estimate the incremental cost per quality-adjusted life-year (QALY) gained of each treatment. This analysis will not consider missed school or work.

429 The parameters needed to populate the models will include outcomes estimated by the NMA of the IPD (e.g., efficacy and safety parameters) and other parameters (e.g., utilities, resource use, and the long-term costs of care) that will require searching of evidence beyond the studies included in the NMA. These will be sourced from a purposive review of the literature, and by using specialist databases (e.g., NHS EED for resource use parameters, and the Tufts CEA registry for utility parameters) where necessary. Unit cost data will be derived from standard national sources (e.g., NHS reference costs[45] and the British National Formulary[46]).

436 A Markov model will be necessary for longer term extrapolation of costs and outcomes, to allow for any differential impacts of treatments over time, and for transition of patients among the 5 steps of treatment.[47] Results of the NMA will be converted to transition probabilities based on standard methods.[48, 49] The model will evaluate costs and outcomes over the lifetime of the patient cohorts: costs and benefits in future years will be discounted at an annual rate of 3.5% and varied between 0% and 6% in sensitivity analysis. Results from the model will be reported as incremental cost per QALY gained (ICERs) and compared with the NICE threshold range of £20,000 to £30,000 per QALY.

For peer review only

444 Uncertainties in all parameter inputs will be accounted for in the analysis by including parametric distributions for each point estimate, supplemented by expert opinion, where the evidence is not sufficiently detailed. This will enable probabilistic sensitivity analyses to be performed based on sampling from distributions using Monte Carlo simulation. The NMA estimates relative effects jointly, and the full joint distribution of these effects will be used in the economic model in order to preserve correlation.[50] Uncertainty in the optimally cost-effective treatment will be represented by cost-effectiveness acceptability curves (CEACs),[51] which present the probability that each drug is the most cost-effective at a given threshold of cost effectiveness. Where the IPD-NMA indicates that patient characteristics modify treatment effects, a stratified approach will be used to assess cost-effectiveness in particular patient sub-group(s). Standard techniques (e.g., extreme value scenarios) will be used to ensure the internal validity of the model.

We will conduct a value of information analysis to inform future research priorities, e.g., whether more short-term efficacy trials are needed, or more long-term follow-up, or more data on the utilities or costs to reduce decision uncertainty.[52] The expected value of perfect information (EVPI) and the expected value of perfect parameter information (EVPPI) will be calculated on both per-patient and population levels using the Sheffield Accelerated Value of Information approximation[53] to facilitate computation effort.

Patient and Public Involvement

The EINSTEIN protocol has been developed in consultation with children with asthma and their parents, and with NHS clinicians who routinely care for children with uncontrolled asthma in NHS settings. Firstly, we have sought advice on our proposal, and the lay summary, from five families, including two children, who attend our asthma clinic at Alder Hey. Secondly, the outcomes chosen in our review have been selected from a core outcome set which were agreed as important by clinicians and patients.[21] This earlier work was heavily influenced by patients and, in particular, highlighted the importance to parents of including mortality and long-term adverse effects as

For peer review only

outcomes. Finally, we consulted an Alder Hey patient advisory group, comprising children with asthma and their parents, and asked them to comment specifically on the lay summary and choice of outcomes in the review project. We will consult with the patient group towards the end of the project to help prepare a patient friendly podcast or leaflet that could be shared on asthma websites and in clinics. We will share our findings at important international meetings, and in medical journals, and we will make sure that this process reflects issues that are important to children and families. Our patient representative (OF) will help to coordinate the group of patients and parents, along with physicians involved. Patients will not be involved in the recruitment to and conduct of the study.

Authors' contributions

CTS is the guarantor. CTS and IS conceived the study. CTS, SD, ST, DH, OF and IS drafted the original version of the protocol, and SC drafted this manuscript. All authors contributed to the development of the selection and data extraction criteria and the strategy of risk of bias assessment. MM developed the search strategy. CTS and SD provided statistical expertise. IS and ST provided clinical expertise. DH and GC contributed to the section on health economics. OF contributed to coordinating the group of patients and parents and developing the plain-language summary. All authors read, provided feedback, and approved the final manuscript.

Funding statement

This work was supported by NIHR HTA grant number 16/110/16. The funder had no role in the protocol development. The views expressed are those of the authors and not necessarily those of the NHS, the NIHR or the Department of Health.

Competing interests statement

The authors declare not to have conflicts of interest.

Acknowledgements

We thank patients and their families for their contribution to this project.

For peer review only

REFERENCES

1 Global Initiative for Asthma (GINA). Global Strategy for Asthma Management and Prevention, 2019. www.ginasthma.org

2 Asthma UK. Access date: February 2020. https://www.asthma.org.uk/about/media/facts-and-statistics/

3 NHS England. Access date: February 2020. https://www.england.nhs.uk/childhood-asthma/

4 British Thoracic Society SIGN. British guideline on the management of asthma. Access Date: February 2020. https://www.brit-thoracic.org.uk/quality-improvement/guidelines/asthma/

5 Asthma: diagnosis, monitoring and chronic asthma management. Access Date: April 2020. https://www.nice.org.uk/guidance/ng80

6 Turner S, Thomas M, von Ziegenweidt J, et al. Prescribing trends in asthma: a longitudinal observational study. *Arch Dis Child* 2009;94(1):16–22. http://dx.doi.org/10.1136/adc.2008.140681

7 Schmier JK, Manjunath R, Halpern MT, et al. The impact of inadequately controlled asthma in urban children on quality of life and productivity. *Ann Allergy Asthma Immunol* 2007;98(3):245–51. https://doi.org/10.1016/S1081-1206(10)60713-2

8 Fuhrman C, Dubus JC, Marguet C, et al. Hospitalizations for asthma in children are linked to undertreatment and insufficient asthma education. *J Asthma* 2011;48(6):565–71. https://doi.org/10.3109/02770903.2011.580031

9 Sala KA, Carroll CL, Tang YS, et al. Factors associated with the development of severe asthma exacerbations in children. *J Asthma* 2011;48(6):558–64. https://doi.org/10.3109/02770903.2011.585411

10 Ungar WJ, Paterson JM, Gomes T, et al. Relationship of asthma management, socioeconomic status, and medication insurance characteristics to exacerbation frequency in children with asthma. *Ann Allergy Asthma Immunol* 2011;106(1):17–23. https://doi.org/10.1016/j.anai.2010.10.006

For peer review only

11 Stelmach I, Podlecka D, Smejda K, et al. Pediatric asthma caregiver's quality of life questionnaire is a useful tool for monitoring asthma in children. *Qual Life Res* 2012;21(9):1639–1642. https://doi.org/10.1007/s11136-011-0070-x

12 Milton B, Whitehead M, Holland P, et al. The social and economic consequences of childhood asthma across the lifecourse: a systematic review. *Child Care Health Dev* 2004;30(6):711–28. https://doi.org/10.1111/j.1365-2214.2004.00486.x

13 Szefler SJ, Phillips BR, Martinez FD, et al. Characterization of within-subject responses to fluticasone and montelukast in childhood asthma. *J Allergy Clin Immunol* 2005;115(2):233–42. https://doi.org/10.1016/j.jaci.2004.11.014

14 Lemanske RF, Mauger DT, Sorkness CA, et al. Step-up Therapy for Children with Uncontrolled Asthma While Receiving Inhaled Corticosteroids. *N Engl J Med* 2010;362(11):975–85. https://doi.org/10.1056/NEJMoa1001278

15 Chauhan BF, Chartrand C, Ni Chroinin M, et al. Addition of long-acting beta2-agonists to inhaled corticosteroids for chronic asthma in children. *Cochrane Database Syst Rev* 2015(11):CD007949. https://doi.org/10.1002/14651858.CD007949.pub2

16 Chauhan BF, Ben Salah R, Ducharme FM. Addition of anti-leukotriene agents to inhaled corticosteroids in children with persistent asthma. *Cochrane Database Syst Rev* 2013(10):CD009585. https://doi.org/10.1002/14651858.CD009585.pub2

17 Zhao Y, Han S, Shang J, et al. Effectiveness of drug treatment strategies to prevent asthma exacerbations and increase symptom-free days in asthmatic children: a network meta-analysis. *J Asthma* 2015;52(8):846–57. https://doi.org/10.3109/02770903.2015.1014101

18 van der Mark LB, Lyklema PH, Geskus RB, et al. A systematic review with attempted network meta-analysis of asthma therapy recommended for five to eighteen year olds in GINA steps three and four. *BMC Pulm Med* 2012;12:63. https://doi.org/10.1186/1471-2466-12-63

19 Hutton J, Williamson P. Bias in meta-analysis with variable selection within studies. *Applied Statistics* 2000;49:359–70. https://doi.org/10.1111/1467-9876.00197

For peer review only

20 Lenney W, McKay AJ, Tudur Smith C, et al. Management of Asthma in School age Children On Therapy (MASCOT): a randomised, double-blind, placebo-controlled, parallel study of efficacy and safety. *Health Technol Assess* 2013;17(4):1–218. https://doi.org/10.3310/hta17040

21 Lenney W, Perry S, Price D. Clinical trials and tribulations: the MASCOT study. *Thorax* 2011;66(6):457–8. https://doi.org/10.1136/thx.2010.156885

22 Moher D, Shamseer L, Clarke M, et al. Preferred reporting items for systematic review and meta-analysis protocols (PRISMA-P) 2015 statement. *Syst rev* 2015;4:1. https://doi.org/10.1186/2046-4053-4-1

23 Sinha IP, Gallagher R, Williamson PR, et al. Development of a core outcome set for clinical trials in childhood asthma: a survey of clinicians, parents, and young people. *Trials* 2012;13:103. https://doi.org/10.1186/1745-6215-13-103

24 Reddel HK, Taylor DR, Bateman ED, et al. An official American Thoracic Society/European Respiratory Society statement: asthma control and exacerbations: standardizing endpoints for clinical asthma trials and clinical practice. *Am J Respir Crit Care Med* 2009;180(1):59–99. https://doi.org/10.1164/rccm.200801-060ST

25 Nathan RA, Sorkness CA, Kosinski M, et al. Development of the asthma control test: a survey for assessing asthma control. *J Allergy Clin Immunol* 2004;113(1):59–65. https://doi.org/10.1016/j.jaci.2003.09.008

26 Juniper EF, O'Byrne PM, Guyatt GH, et al. Development and validation of a questionnaire to measure asthma control. *Eur Respir J* 1999;14:902–7. https://doi.org/10.1034/j.1399-3003.1999.14d29.x

27 Juniper EF, Svensson K, Mörk AC, et al. Measurement properties and interpretation of three shortened versions of the asthma control questionnaire. *Respir Med* 2005;99:553–8. https://doi.org/10.1016/j.rmed.2004.10.008

For peer review only

28 Juniper EF, Bousquet J, Abetz L, et al. Identifying ‘well-controlled’ and ‘not well-controlled’ asthma using the Asthma Control Questionnaire. *Respir Med* 2006;100:616–621. https://doi.org/10.1016/j.rmed.2005.08.012

29 Juniper EF, Gruffydd-Jones K, Ward S, et al. Asthma Control Questionnaire in Children: Validation, Measurement Properties, Interpretation. *Eur Respir J* 2010;36:1410–1416. https://doi.org/10.1183/09031936.00117509

30 Chauhan BF, Ducharme FM. Addition to inhaled corticosteroids of long-acting beta2-agonists versus anti-leukotrienes for chronic asthma. *Cochrane Database Syst Rev* 2014(1):CD003137. https://doi.org/10.1002/14651858.CD003137.pub5

31 Chauhan BF, Ducharme FM. Anti-leukotriene agents compared to inhaled corticosteroids in the management of recurrent and/or chronic asthma in adults and children. *Cochrane Database Syst Rev* 2012(5):CD002314. https://doi.org/10.1002/14651858.CD002314.pub3

32 Pruteanu AI, Chauhan BF, Zhang L, et al. Inhaled corticosteroids in children with persistent asthma: dose-response effects on growth. *Cochrane Database Syst Rev* 2014(7):CD009878. https://doi.org/10.1002/14651858.CD009878.pub2

33 Tudur Smith C, Hopkins C, Sydes MR, et al. How should individual participant data (IPD) from publicly funded clinical trials be shared? *BMC Med* 2015;13(1):1–7. https://doi.org/10.1186/s12916-015-0532-z

34 Higgins JPT, Altman DG, Gøtzsche PC, et al. The Cochrane Collaboration’s tool for assessing risk of bias in randomised trials. *BMJ* 2011;343:d5928. https://doi.org/10.1136/bmj.d5928

35 The transitivity assumption CJC. Available from: https://airways.cochrane.org/sites/airways.cochrane.org/files/public/uploads/The transitivity assumption CJC.pdf

36 Dias S, Welton NJ, Caldwell DM, et al. Checking consistency in mixed treatment comparison meta-analysis. *Stat Med* 2010;29(7-8):932–44. https://doi.org/10.1002/sim.3767

For peer review only

37 Donegan S, Williamson P, D'Alessandro U, et al. Assessing key assumptions of network meta-analysis: a review of methods. *Res Synth Methods* 2013;4(4):291–323. https://doi.org/10.1002/jrsm.1085

38 Donegan S, Williamson P, D'Alessandro U, et al. Combining individual patient data and aggregate data in mixed treatment comparison meta-analysis: Individual patient data may be beneficial if only for a subset of trials. *Stat Med* 2013;32(6):914–30. https://doi.org/10.1002/sim.5584

39 Cooper NJ, Sutton AJ, Morris D, et al. Addressing between-study heterogeneity and inconsistency in mixed treatment comparisons: Application to stroke prevention treatments in individuals with non-rheumatic atrial fibrillation. *Stat Med* 2009;28(14):1861–81. https://doi.org/10.1002/sim.3594

40 Saramago P, Sutton AJ, Cooper NJ, et al. Mixed treatment comparisons using aggregate and individual participant level data. *Stat Med* 2012;31(28):3516–36. https://doi.org/10.1002/sim.5442

41 Donegan S, Williamson P, D'Alessandro U, et al. Assessing the consistency assumption by exploring treatment by covariate interactions in mixed treatment comparison meta-analysis: individual patient-level covariates versus aggregate trial-level covariates. *Stat Med* 2012;31(29):3840–57. https://doi.org/10.1002/sim.5470

42 Petrou S, Gray A. Economic evaluation using decision analytical modelling: design, conduct, analysis, and reporting. *BMJ* 2011;342:d1766. https://doi.org/10.1136/bmj.d1766

43 Husereau D, Drummond M, Petrou S, et al. Consolidated Health Economic Evaluation Reporting Standards (CHEERS) statement. *BMJ* 2013;346:f1049. https://doi.org/10.1136/bmj.f1049

44 National Institute for Health and Care Excellence. Guide to the methods of technology appraisal 2013. Available from: https://www.nice.org.uk/process/pmg9

45 NHS Improvement. NHS Reference costs 2017/18. https://improvement.nhs.uk/documents/6468/201718_reference_costs_data_and_guidance.zip

46 British National Formulary 79. March-September 2020

For peer review only

47 Ehteshami-Afshar S, Zafari Z, Hamidi N, et al. A Systematic Review of Decision-Analytic Models for Evaluating Cost-Effectiveness of Asthma Interventions. *Value Health* 2019;22(9):1070–1082. https://doi.org/10.1016/j.jval.2019.03.016

48 Gidwani R, Russell LB. Estimating Transition Probabilities from Published Evidence: A Tutorial for Decision Modelers. *Pharmacoeconomics* 2020;38(11):1153–1164. https://doi.org/10.1007/s40273-020-00937-z

49 Welton NJ, Sutton AJ, Cooper NJ, et al. Evidence Synthesis for Decision Making in Healthcare, John Wiley & Sons, Ltd. 2012.

50 Dias S, Sutton AJ, Welton NJ, et al. Evidence synthesis for decision making 6: embedding evidence synthesis in probabilistic cost-effectiveness analysis. *Med Decis Making* 2013;33(5):671-8. https://doi.org/10.1177/0272989X13487257

51 Fenwick E, Claxton K, Sculpher M. Representing uncertainty: the role of cost-effectiveness acceptability curves. *Health Econ* 2001;10(8):779-87. https://doi.org/10.1002/hec.635

52 Claxton KP, Sculpher MJ. Using value of information analysis to prioritise health research: some lessons from recent UK experience. *Pharmacoeconomics* 2006;24(11):1055-68. https://doi.org/10.2165/00019053-200624110-00003

53 Strong M, Oakley JE, Brennan A. Estimating multi-parameter partial expected value of perfect information from a probabilistic sensitivity analysis sample: a non-parametric regression approach. *Med Decis Making* 2014;34(3):311-26. https://doi.org/10.1177/0272989X13505910

Figure caption

Figure 1 Treatment step-up for asthmatic patients according to age group. Adapted from GINA 2019.[1] SABA = Short-acting β_2-adrenoceptor agonist; ICS = Inhaled corticosteroid; LTRA = Leukotriene receptor antagonist; LABA = Long-acting β_2-adrenoceptor agonist; OCS = Oral corticosteroids ; IgE = Immunoglobulin E; IL = Interleukin

For peer review only

≤ 5-year-old (Children)

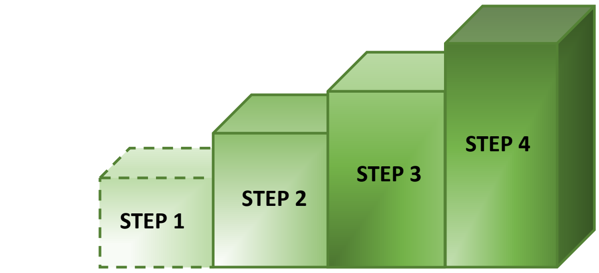

STEP 1 SABA as needed
STEP 2 Daily low dose ICS – SABA as needed.
Other controller options: LTRA, or intermittent ICS.
STEP 3 Double low dose ICS – SABA as needed
Other controller options: low dose ICS + LTRA.
STEP 4 Continue controller + specialist assessment
Other controller options: add LTRA / increase ICS frequency / add intermittent ICS.

Reliever: as-needed SABA

6-11-year-old (Children)

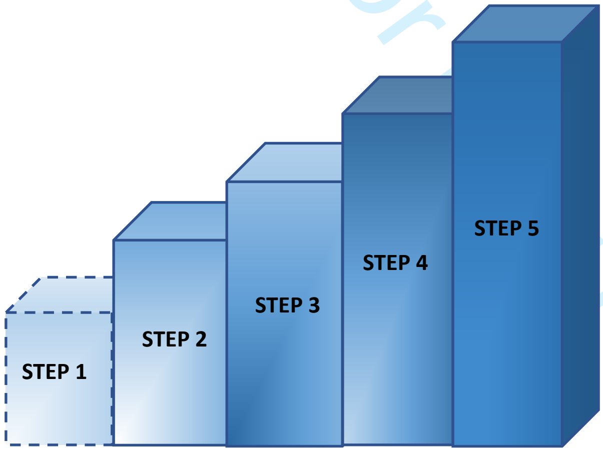

STEP 1 SABA as needed.
Other controller options: low dose of ICS taken with SABA, or daily low dose ICS.
STEP 2 Daily low dose ICS – SABA as needed.
Other controller options: LTRA, or low dose ICS taken with SABA.
STEP 3 Low dose ICS+LABA, or medium dose ICS – SABA as needed. *Other controller options*: low dose ICS+LTRA.
STEP 4 Medium dose ICS+LABA – SABA as needed.
Other controller options: high dose ICS+LABA, or add-on tiotropium, or add-on LTRA.
STEP 5 Phenotypic assessment ± add-on therapy (e.g., anti-IgE) – SABA as needed. *Other controller options*: add-on anti-IL5, or add-on low dose OCS.

Reliever: as-needed SABA

≥ 12-year-old (Adults and adolescents)

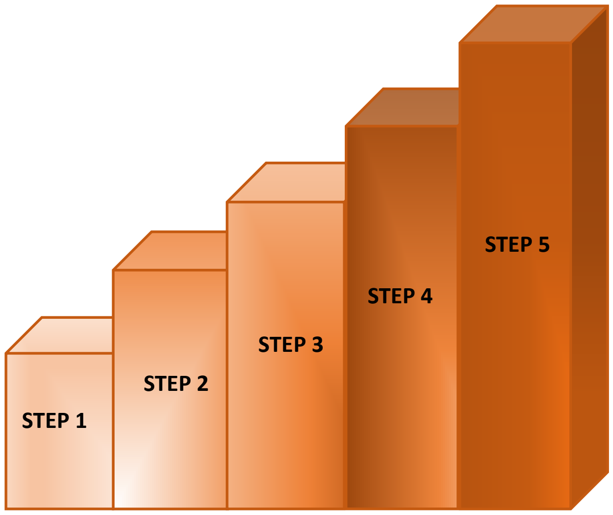

STEP 1 Low dose ICS/formoterol as needed.
Other controller options: low dose of ICS taken with SABA.
STEP 2 Daily low dose ICS – Low dose ICS/formoterol as needed. *Other controller options*: LTRA, or low dose ICS taken with SABA.
STEP 3 Low dose ICS+LABA – Low dose ICS/Formoterol as needed. *Other controller options*: medium dose ICS, or low dose ICS+LTRA.
STEP 4 Medium dose ICS+LABA – Low dose ICS/Formoterol as needed. *Other controller options*: high dose ICS, add-on tiotropium, or add-on LTRA.
STEP 5 High dose ICS+LABA. Phenotypic assessment ± add-on therapy (e.g., tiotropium, anti-IgE, anti-IL5/5R, anti-IL4R) – Low dose ICS/Formoterol as needed.
Other controller options: add-on low dose OCS.

Preferred reliever: as-needed low dose ICS/formoterol
Reliever option: as-needed SABA

For peer review only

EstablishINg the best STEp-up treatments for children with uncontrolled asthma despite INhaled corticosteroids (EINSTEIN): Protocol for a systematic review, network meta-analysis and cost effectiveness analysis using individual participant data (IPD)

Sofia Cividini[1], Ian Sinha[2], Sarah Donegan[1], Michelle Maden[3], Giovanna Culeddu[4], Katie Rose[2], Olive Fulton[*], Dyfrig A. Hughes[4], Stephen Turner[5], Catrin Tudur Smith[1]

[1]Department of Biostatistics, University of Liverpool, Liverpool, UK
[2]Alder Hey Children's Foundation NHS Trust, Liverpool, UK
[3]Liverpool Reviews and Implementation Group (LRIG), University of Liverpool, Liverpool, UK
[4]Centre for Health Economics & Medicines Evaluation, Bangor University, Bangor, UK
[5]University Court of the University of Aberdeen, Aberdeen, UK
[*]Patients Representative, Liverpool, UK

For peer review only

Appendix 1. Example MEDLINE (OVID) search

1 exp Asthma/
2 asthma.ti,ab.
3 1 or 2
4 exp Infant/
5 infant*.ti,ab.
6 infancy.ti,ab.
7 newborn*.ti,ab.
8 baby*.ti,ab.
9 babies.ti,ab.
10 neonat*.ti,ab.
11 preterm*.ti,ab.
12 prematur*.ti,ab.
13 postmatur*.ti,ab.
14 exp child/
15 child*.ti,ab.
16 schoolchild*.ti,ab.
17 "school age*".ti,ab.
18 preschool*.ti,ab.
19 kid.ti,ab.
20 kids.ti,ab.
21 toddler*.ti,ab.
22 exp Adolescent/
23 adoles*.ti,ab.
24 teen*.ti,ab.
25 boy*.ti,ab.
26 girl*.ti,ab.
27 exp Minors/
28 minor*.ti,ab.
29 exp Puberty/
30 pubert*.ti,ab.
31 pubescen*.ti,ab.
32 prepubescen*.ti,ab.
33 exp Pediatrics/
34 paediatric*.ti,ab.
35 pediatric*.ti,ab.

36 exp Schools/
37 "nursery school*".ti,ab.
38 kindergar*.ti,ab.
39 "primary school*".ti,ab.
40 "secondary school*".ti,ab.
41 "elementary school*".ti,ab.
42 "high school*".ti,ab.
43 highschool*.ti,ab.
44 or/4-43
45 "inhaled corticosteroid*".mp.
46 ICS.mp.
47 exp Beclomethasone/
48 beclomethasone.mp.
49 "beclomethasone dipropionate".mp.
50 becotide.mp.
51 clenil.mp.
52 ciclesonide.mp.
53 "clenil modulite".mp.
54 exp Fluticasone/
55 "fluticasone propionate".mp.
56 fluticasone.mp.
57 flixotide.mp.
58 exp Budesonide/
59 budesonide.mp.
60 Mometasone Furoate/
61 mometasone.mp.
62 exp Adrenergic beta-Agonists/
63 "long acting beta-2 agonist*".mp.
64 "long acting beta2 agonist*".mp.
65 LABA.mp.
66 exp Formoterol Fumarate/
67 formoterol.mp.
68 Oxis.mp.
69 "fluticasone furoate".mp.
70 exp Salmeterol Xinafoate/
71 salmeterol.mp.
72 serevent.mp.
73 vilanterol.mp.
74 exp Leukotriene Antagonists/
75 "leukotriene receptor antagonist*".mp.
76 LTRA.mp.
77 zafirlukast.mp.
78 montelukast.mp.
79 exp Theophylline/
80 theophylline.mp.
81 Tiotropium.mp.
82 spiriva.mp.
83 Symbicort.mp.
84 Seretide.mp.
85 flutiform.mp.
86 relvar.mp.
87 or/45-86
88 Clinical Trial.pt.

For peer review only

3

89 Randomized Controlled Trial.pt.
90 exp Random Allocation/
91 exp Single-Blind Method/
92 exp Double-Blind Method/
93 exp Cross-Over Studies/
94 exp Placebos/
95 RCT.ti,ab.
96 Random*.ti,ab.
97 "Single blind*".ti,ab.
98 "Double blind*".ti,ab.
99 "triple blind*".ti,ab.
100 placebo*.ti,ab.
101 or/88-100
102 3 and 44 and 87 and 101
103 limit 102 to ed=20140701-20190911
104 limit 103 to english language
105 (case reports or editorial or letter).pt.
106 104 not 105

For peer review only

BMJ Open

EstablishINg the best STEp-up treatments for children with uncontrolled asthma despite INhaled corticosteroids (EINSTEIN): Protocol for a systematic review, network meta-analysis and cost-effectiveness analysis using individual participant data (IPD)

Journal:	*BMJ Open*
Manuscript ID	bmjopen-2020-040528.R2
Article Type:	Protocol
Date Submitted by the Author:	16-Dec-2020

Complete List of Authors:	Cividini, Sofia; University of Liverpool, Biostatistics<br>Sinha, Ian; Alder Hey Children's NHS Foundation Trust<br>Donegan, Sarah; University of Liverpool, Biostatistics<br>Maden, Michelle; University of Liverpool, Liverpool Reviews and Implementation Group<br>Culeddu, Giovanna; Bangor University, Centre for Health Economics and Medicines Evaluation<br>Rose, Katie; Alder Hey Children's NHS Foundation Trust<br>Fulton, Olive; Patients Representative<br>Hughes, Dyfrig; Bangor University, Centre for Health Economics and Medicines Evaluation<br>Turner, Stephen; University Court of the University of Aberdeen, Department of Child Health<br>Tudur-Smith, Catrin; University of Liverpool, Biostatistics

**Primary Subject Heading**:	Respiratory medicine
Secondary Subject Heading:	Epidemiology, Evidence based practice, Health economics, Paediatrics, Research methods
Keywords:	STATISTICS & RESEARCH METHODS, Asthma < THORACIC MEDICINE, Epidemiology < THORACIC MEDICINE, PAEDIATRICS

SCHOLARONE™
Manuscripts

I, the Submitting Author has the right to grant and does grant on behalf of all authors of the Work (as defined in the below author licence), an exclusive licence and/or a non-exclusive licence for contributions from authors who are: i) UK Crown employees; ii) where BMJ has agreed a CC-BY licence shall apply, and/or iii) in accordance with the terms applicable for US Federal Government officers or employees acting as part of their official duties; on a worldwide, perpetual, irrevocable, royalty-free basis to BMJ Publishing Group Ltd ("BMJ") its licensees and where the relevant Journal is co-owned by BMJ to the co-owners of the Journal, to publish the Work in this journal and any other BMJ products and to exploit all rights, as set out in our licence.

The Submitting Author accepts and understands that any supply made under these terms is made by BMJ to the Submitting Author unless you are acting as an employee on behalf of your employer or a postgraduate student of an affiliated institution which is paying any applicable article publishing charge ("APC") for Open Access articles. Where the Submitting Author wishes to make the Work available on an Open Access basis (and intends to pay the relevant APC), the terms of reuse of such Open Access shall be governed by a Creative Commons licence – details of these licences and which Creative Commons licence will apply to this Work are set out in our licence referred to above.

Other than as permitted in any relevant BMJ Author's Self Archiving Policies, I confirm this Work has not been accepted for publication elsewhere, is not being considered for publication elsewhere and does not duplicate material already published. I confirm all authors consent to publication of this Work and authorise the granting of this licence.

For peer review only

EstablishINg the best STEp-up treatments for children with uncontrolled asthma despite INhaled corticosteroids (EINSTEIN): Protocol for a systematic review, network meta-analysis and cost-effectiveness analysis using individual participant data (IPD)

Sofia Cividini[1], Ian Sinha[2§], Sarah Donegan[1], Michelle Maden[3], Giovanna Culeddu[4], Katie Rose[2], Olive Fulton[*], Dyfrig A. Hughes[4], Stephen Turner[5], Catrin Tudur Smith[1§**]

[1]Department of Biostatistics, University of Liverpool, Liverpool, UK

Catrin Tudur Smith – Institute of Translational Medicine, Department of Biostatistics, University of Liverpool, Crown Street, Liverpool L693BX, UK – cat1@liverpool.ac.uk – Tel. +44 (0)151 794 4059/+44 (0)151 794 9754 – ORCID: https://orcid.org/0000-0003-3051-1445

Sofia Cividini – Sofia.Cividini@liverpool.ac.uk – ORCID: https://orcid.org/0000-0003-2705-9224

Sarah Donegan – Sarah.Donegan@liverpool.ac.uk – ORCID: https://orcid.org/0000-0003-1709-2290

[2]Alder Hey Children's Foundation NHS Trust, Liverpool, UK

Ian Sinha – Ian.Sinha@alderhey.nhs.uk – ORCID: https://orcid.org/0000-0002-7342-5523

Katie Rose – katierose100@gmail.com – ORCID: https://orcid.org/0000-0002-2348-2036

[3]Liverpool Reviews and Implementation Group (LRIG), University of Liverpool, Liverpool, UK

Michelle Maden – mmaden@liverpool.ac.uk – ORCID: https://orcid.org/0000-0003-4419-6343

[4]Centre for Health Economics & Medicines Evaluation, Bangor University, Bangor, UK

Dyfrig Hughes – d.a.hughes@bangor.ac.uk – ORCID: https://orcid.org/0000-0001-8247-7459

Giovanna Culeddu – g.culeddu@bangor.ac.uk – ORCID: https://orcid.org/0000-0001-5032-4255

[5]University Court of the University of Aberdeen, Aberdeen, UK

Stephen Turner – s.w.turner@abdn.ac.uk – ORCID: https://orcid.org/0000-0001-8393-5060

[*]Patients Representative, Liverpool, UK

Olive Fulton – olive.fulton@gmail.com

Running head: The EINSTEIN Study Protocol (IPD-NMA)

[**]Correspondence to Professor Catrin Tudur Smith; cat1@liverpool.ac.uk

[§]CTS and IS are joint senior authors

ABSTRACT

Introduction

Asthma affects millions of children worldwide – 1.1 million children in the UK. Asthma symptoms cannot be cured but can be palliated with low dose inhaled corticosteroids (ICS) in the majority of individuals. Treatment with a low dose ICS, however, fails to control asthma symptoms in around 10-15% of children and this places the individual at increased risk for an asthma attack. At present, there is no clear preferred treatment option for a child whose asthma is not controlled by low dose ICS, and international guidelines currently recommend at least three treatment options. Herein we propose a systematic review and individual participant data network meta-analysis (IPD-NMA)

For peer review only

aiming to synthesise all available published and unpublished evidence from randomised controlled trials (RCTs) to establish the clinical effectiveness of pharmacological treatments in children and adolescents with uncontrolled asthma on ICS and help to make evidence-informed treatment choices. This will be used to parameterise a Markov-based economic model to assess the cost-effectiveness of alternative treatment options in order to inform decisions in the context of drug formularies and clinical guidelines.

Methods and analysis

We will search in MEDLINE, the Cochrane Library, the Cochrane Central Register of Controlled Trials (CENTRAL), Embase, NICE Technology Appraisals, and the NIHR HTA series for RCTs of interventions in patients with uncontrolled asthma on ICS. All studies where children and adolescents were eligible for inclusion will be considered, and authors or sponsors will be contacted to request IPD on patients aged <18. The reference lists of existing clinical guidelines, along with included studies and relevant reviews, will be checked to identify further relevant studies. Unpublished studies will be located by searching across a range of clinical trial registries, including internal trial registers for pharmaceutical companies. All studies will be appraised for inclusion against predefined inclusion and exclusion criteria by two independent reviewers with disagreements resolved through discussion with a third reviewer. We will perform an IPD-NMA – eventually supplemented with aggregate data for the RCTs without IPD – to establish both the probability that a treatment is best and the probability that a particular treatment is most likely to be effective for a specific profile of the patient. The IPD-NMA will be performed for each outcome variable within a Bayesian framework, using the WinBUGS software. Also, potential patient-level characteristics that may modify treatment effects will be explored, which represents one of the strengths of this study.

Ethics and dissemination

The University of Liverpool committee on Research Ethics has confirmed that ethics review is not required. The dissemination plan consists of publishing the results in an open-access medical

For peer review only

journal, a plain-language summary available for parents and children, dissemination via local, national, and international meetings and conferences, and the Press Offices of our HEIs. A synopsis of results will be disseminated to NICE and BTS/SIGN as highly relevant to future clinical guideline updates.

PROSPERO registration number: CRD42019127599 – This project is funded by the NIHR Health Technology Assessment Programme (project number 16/110/16).

Keywords inhaled corticosteroids ▪ long-acting β_2-agonists ▪ leukotriene receptor antagonist ▪ theophylline ▪ exacerbation ▪ asthma control ▪ hospital admissions ▪ adolescents

Strengths and limitations of this study

- This study will be the most comprehensive synthesis of interventional studies of children and adolescents with uncontrolled asthma on ICS.
- It will be able to establish the most clinically and cost-effective step-up therapy for asthma, also considering those treatments where no head-to-head comparison exists, providing evidence to make informed treatment choices.
- It will be able to evaluate the influence of patient-level characteristics on treatment effect.
- It will provide an assessment of the incremental cost per quality-adjusted life-year gained of treatment options, from the perspective of the NHS and personal social services.
- Individual participant data may not be available from all eligible RCTs and integration with aggregate data may be needed.

INTRODUCTION

Rationale

Recent epidemiological data show that an always-increasing number of people of all age group worldwide, among that millions of children and adolescents, are affected by asthma with a notable social and economic burden for health systems.[1] Asthma remains a common medical condition affecting over one million children in the UK, one of the highest prevalence rate worldwide.[2, 3]

For peer review only

Asthma is characterised by symptoms of wheeze, breathlessness, chest-tightness, cough and can affect the child's quality of life by limiting daily activities and causing acute attacks. In the UK, it was estimated that one child is admitted to the hospital every 20 minutes because of an asthma attack.[2]

The British Guideline[4, 5] on the management of asthma recommends that, following a diagnosis of asthma in a child, a stepwise approach to treatment should be taken. The initial level of treatment is a low dose inhaled corticosteroid to prevent symptoms plus a short acting β_2-adrenoceptor agonist (SABA) to relieve symptoms. However, treatment with a low dose ICS fails to control asthma symptoms in around 10-15% of children.[6] If asthma remains uncontrolled, a series of further steps are followed consisting of a treatment step-up by including add-on preventer therapies such as long-acting β_2-adrenoceptor agonists (LABA) or leukotriene receptor antagonists (LTRA), increasing the dose of ICS, or adding sustained-release (SR) theophylline.[1, 4, 5]

Choosing the best step-up treatment thus becomes a crucial decision to prevent occurring of exacerbations and to avoid poor asthma control, also improving the quality of life of patients and their families and optimising the use of NHS resources.[7-12] At present, no clear preferential option for initial step-up exists, and the decision-making process about treatment choices mainly bases on clinicians' preferences and not on evidence-based practice. Moreover, there is substantial heterogeneity among individuals in the treatment response.[13, 14]

A recent Cochrane review[15] comprising of 33 trials with 6,381 children demonstrated that adding LABAs to ICSs was not associated with a significant decrease in the rate of exacerbations requiring systemic steroids but was superior for improving lung function (e.g., FEV_1 and PEF), compared with the same or higher doses of ICS. No statistically significant group difference in hospital admissions was found. Notwithstanding, because of a trend towards increased risk of hospital admission with LABA independently of ICS dose, the authors advised further monitoring of treatment.[15]

For peer review only

Another Cochrane review[16] published in 2013 found that adding LABAs to ICSs was associated with no statistically significant reduction neither of the use of oral corticosteroids as rescue nor hospital admissions compared to the same or an increased dose of ICS. Evidence was based only on 4 trials with 559 children and adolescents having mild to moderate asthma.[16] The authors cautioned that the lack of paediatric trials, the absence of data on pre-schoolers, and the variability in the reporting of relevant clinical outcomes was a limit for firm conclusions regarding this comparison.[16]

Two network meta-analyses (NMAs), analysing published RCT-level aggregate data for children with uncontrolled asthma, have already been conducted,[17, 18] but the evidence from these analyses has important limitations. The considerable variation in, and the incomplete reporting of outcome measurements across RCTs prevented a formal NMA and assessment of relative efficacies of treatments in one NMA of 23 RCTs (4,129 patients).[18] In 2015, Zhao et al.[17] conducted a formal NMA consisting of 35 RCTs with 12,010 children. These authors suggested that combined ICS and LABA treatments were most effective in preventing exacerbations and that treatments with medium- or high-dose ICS, combined ICS/LTRA, and low-dose ICS seem to be equally effective.[17] However, the analysis has limitations as the authors excluded 70 relevant RCTs because publications of these RCTs did not provide data about 'exacerbations' or 'symptom-free days'. Outcome reporting bias[19] is, therefore, a severe threat to the validity of their results if the excluded studies had selectively reported results based on the statistical significance of their findings. Moreover, a series of other issues make the interpretation and generalisability of the results of this NMA difficult. There is a lack of complete overlapping of the included studies compared to the previous Cochrane reviews[15, 16] and previous planned NMA[18] – only 7 RCTs are in common across the two NMAs.[17, 18] Two other weak points are represented by the lack of analyses focussing on outcomes of importance and relevance to patients and the lack of analyses comparing different drugs (ICS, LABA, LTRA), doses, and types of inhalation devices within ICS

For peer review only

and LABA classes. We have also included tiotropium in the search algorithm as a long-acting antimuscarinic medication (LAMA), but we are not confident of finding sufficient studies.

Several randomised clinical trials (RCTs) have compared two alternative step-up options head-to-head, but only a few individual trials have analysed more than two classes head-to-head. The BADGER trial[14] randomised 182 children and adolescents with uncontrolled asthma on fluticasone to receive each of the following therapies: 'ICS step-up', 'LABA step-up', and 'LTRA step-up', obtaining differential responses to every step-up treatment. 'LABA step-up' showed significantly more likely to provide the 'best' response; yet, many patients better replied with 'ICS or LTRA step-up' suggesting that for some patients, these treatments may be preferred.[14] Overall, results from BADGER trial were heterogeneous, and a 16-week follow-up may not be sufficient.

The MASCOT trial[20] randomised children aged 6-14 to fluticasone plus placebo, fluticasone/salmeterol plus placebo, and fluticasone plus montelukast. However, this RCT failed to recruit an adequate number of patients due to significant organisational challenges, issues of accessing patients from primary care, and changes in prescribing habits during the trial. These challenges limit the potential success of similar future clinical trials, and recommendations have been made for the use of alternative study designs[20, 21] to compare alternative step-up treatments.

There is thus an urgent need for a novel approach to be taken to synthesise all of the available evidence using robust, unbiased methods, also including unpublished data. A key strength of the EINSTEIN analysis will be the assessment of individual patient-level characteristics in modifying the patient's response to treatment. No study has previously considered this aspect, although it represents a crucial clinical step in evaluating treatment efficacy. Furthermore, an economic analysis of the treatment options will provide evidence on their cost-effectiveness from the perspective of the National Health Service in the UK. Results from the EINSTEIN study will provide clinicians and patients with accessible, high-quality, patient-relevant information to help make evidence-informed treatment choices. Earlier identification of the best step-up treatment for a

For peer review only

particular child could have a significant impact on children's lives with more extensive benefits to society and the NHS.

Objectives

Our research objective is to establish the best treatment option for patients younger than 18 whose asthma symptoms are uncontrolled by low-medium dose ICS. To this goal, the EINSTEIN study will identify and synthesise all available evidence from RCTs to establish the effectiveness of pharmacological treatments in children and adolescents aged <18 having poor asthma control on ICS. The study will also investigate the modifiers of treatment effect (e.g., age, ethnicity, asthmatic phenotype) to optimise a targeted therapy and maximise patients' informed choice of treatment. The economic analysis will estimate the cost-effectiveness of treatments in terms of their incremental costs per quality-adjusted life-year gained.

Specifically, we aim to:

1. Conduct a systematic review to identify relevant RCTs of treatment with children and adolescents whose asthma remains uncontrolled despite ICS use.
2. Collect individual participant data (IPD) from all eligible trials.
3. Carry out a network meta-analysis of IPD to identify the most effective treatment.
4. Identify modifiers of treatment effect to establish which patients respond better to each treatment.
5. Develop an economic model to estimate the incremental cost per quality-adjusted life-year gained of treatment options, from the perspective of the NHS and Personal Social Services.
6. Identify remaining uncertainties formulating recommendations to inform priorities aimed at future research.
7. Disseminate findings.

METHODS

For peer review only

The PRISMA-P guidelines (Preferred Reporting Items for Systematic Reviews and Meta-Analysis Protocols)[22] have guided the drafting of this protocol.

Eligibility criteria

For inclusion in the EINSTEIN study, we will select studies according to the criteria outlined below.

Study designs

We will include parallel and cross-over randomised controlled trials (RCTs), of any duration, using any level of blinding and comparing at least one of the health technologies of interest. All RCTs meeting our inclusion criteria will be included irrespective of the outcomes reported in the publication, to reduce the potential for outcome reporting bias.

Participants

We will focus on children (<12 years) and adolescents (12-17 years) of any ethnicity with poor asthma control on ICS before randomisation as defined by the study protocol. We will include studies with mixed age groups in which children and adolescents were eligible because we will contact authors concerning specific data on patients aged <18. Patients aged ≥18, or with stable asthma as defined by the study authors, or using an asthma medication other than ICS, or an ICS with an add-on active (e.g., ICS/LABA) as treatment regimen before randomisation will not be considered. Participants using any dose ICS at randomisation will be eligible for inclusion.

Interventions

We will consider as eligible all studies where children and adolescents were randomised to at least one of the following treatments:

▪ Inhaled Corticosteroids (ICS), alone or in combination with other treatments – beclometasone dipropionate; ciclesonide; fluticasone propionate; fluticasone furoate; budesonide; mometasone.

▪ Long-acting β_2-agonists (LABA) – formoterol; salmeterol; vilanterol.

▪ Leukotriene receptor antagonists (LTRA) – zafirlukast; montelukast.

For peer review only

▪ Theophylline.

Any dose of preventer treatment – oral or inhaled, and any inhaler devices (pressurised metered-dose inhaler, dry powder inhaler, combination inhaler) – will be considered. Presence or absence of spacer devices will be noted.

We will not consider studies with participants randomised to any asthma medication other than those listed in the inclusion criteria

Comparators

For both primary and secondary outcomes – if not specified otherwise – patient outcomes will be compared at two levels, namely among:

1. Every class of treatment – ICS (alone or with add-on actives), LABA, LTRA, theophylline, and placebo. For ICS, we will distinguish between low, medium, and high doses as defined in GINA – Global Initiative for Asthma 2019[1] (Table 1 and Figure 1).

Table 1 Estimated clinical comparability daily doses (µg) of Inhaled Corticosteroids (ICS)

**≤ 5-year-old (Children)**			
**Drug**	**Low Dose**	**Medium Dose**	**High Dose**
Beclomethasone dipropionate (HFA)	100 (≥5 years)	N.A.	N.A.
Budesonide nebulised	500 (≥1 year)	N.A.	N.A.
Budesonide pMDI + spacer	N.A.	N.A.	N.A.
Fluticasone propionate (HFA)	50 (≥4 years)	N.A.	N.A.
Mometasone furoate	110 (≥4 years)	N.A.	N.A.
Ciclesonide	N.A.	N.A.	N.A.
**6-11-year-old (Children)**			
**Drug**	**Low Dose**	**Medium Dose**	**High Dose**
Beclomethasone dipropionate (CFC)	100-200	>200-400	>400
Beclomethasone dipropionate (HFA)	50-100	>100-200	>200
Budesonide (DPI)	100-200	>200-400	>400
Budesonide (nebules)	250-500	>500-1000	>1000
Ciclesonide	80	>80-160	>160
Fluticasone furoate (DPI)	N.A.	N.A.	N.A.
Fluticasone propionate (DPI)	100-200	>200-400	>400
Fluticasone propionate (HFA)	100-200	>200-500	>500
Mometasone furoate	110	≥220-<440	≥440
**≥ 12-year-old (Adults and adolescents)**			
**Drug**	**Low Dose**	**Medium Dose**	**High Dose**
Beclomethasone dipropionate (CFC)	200-500	>500-1000	>1000
Beclomethasone dipropionate (HFA)	100-200	>200-400	>400
Budesonide (DPI)	200-400	>400-800	>800
Ciclesonide (HFA)	80-160	>160-320	>320

For peer review only

Fluticasone furoate (DPI)	100	N.A.	200
Fluticasone propionate (DPI)	100-250	>250-500	>500
Fluticasone propionate (HFA)	100-250	>250-500	>500
Mometasone furoate	110-220	>220-440	>440

CFC = chlorofluorocarbon propellant (no longer used; included for comparison with older literature)
DPI = dry powder inhaler
HFA = hydrofluoroalkane propellant
N.A. = not applicable

2. Every drugs within every class of treatment – beclometasone dipropionate; ciclesonide; fluticasone propionate; fluticasone furoate; budesonide; mometasone; formoterol; salmeterol; vilanterol; zafirlukast; montelukast; theophylline; (placebo).

Moreover, we will explore complex hierarchical models that consider both classes of treatments and actives with interactions of covariates. Healthy controls will not be considered as an appropriate reference group.

Outcomes

Review outcomes will include the outcomes identified as important to healthcare practitioners and patients during the development of a core outcome set for trials in children with asthma.[23, 24]

Primary outcomes:

- Exacerbations as defined by ERS/ATS.[24]
- Asthma control measured by a validated test (e.g., ACT,[25] ACQ,[26-29] other).

Secondary outcomes:

- Symptoms/symptom score
- Quality of Life (QoL)
- Mortality – although rare, this is an important outcome for parents
- Physiological outcomes (e.g., FEV_1, bronchial responsiveness)
- Adverse effects (AEs) – including growth and withdrawals due to adverse effects
- Hospital admissions
- Total health care costs (obtained by the sum-product of item of resource use and their unit cost), resource use, and utility outcomes to inform the economic model.

For peer review only

Timing

Studies will be selected for inclusion independently of the length of follow-up of outcomes.

Setting

There will be no restrictions by type of setting.

Language

We will include only articles reported in the English language.

Information sources and search strategy

Our search strategy will identify published and unpublished studies and builds upon previously published search strategies for aggregate data network meta-analyses[17, 18] and Cochrane reviews.[15, 16, 30-32] (see Supplementary File 1; for example, MEDLINE search). The search strategies will run from 2014 onwards only. From inception to 2013, we will consider as exhaustive the searches already carried out for the meta-analyses and Cochrane reviews above mentioned,[15-18, 30-32] and we will retrieve all the corresponding studies.

We will search MEDLINE, the Cochrane Library, the Cochrane Central Register of Controlled Trials (CENTRAL), Embase, NICE Technology Appraisals, and the NIHR HTA series using relevant search terms. We will also scan the reference list of included studies and relevant reviews along with the reference lists of existing clinical guidelines such as the British Thoracic Society (BTS) Guideline[4, 5] and GINA.[1]

For unpublished studies, we will search a range of clinical trial registries enclosed inside the WHO 'International Clinical Trials Registry Platform' search portal (including clinicaltrials.gov and ISRCTN) and conference abstracts (e.g., European Respiratory Society; American Thoracic Society).

We will also search internal clinical trial registers for pharmaceutical companies that manufacture health technologies of interest (e.g., GSK, AstraZeneca, Novartis, Merck).

For peer review only

We will limit the search to English language articles, and we will verify that RCTs have included participants aged <18 as a subset.

STUDY RECORDS

Data management

Studies identified for screening of abstracts and full-text articles will be managed within Covidence (https://www.covidence.org/home), a systematic review management tool.

Selection process

All studies will be screened and assessed for inclusion by two independent reviewers (SC and KR) with disagreements resolved through discussion with a third reviewer (IS). To minimise the impact of selective outcome reporting, we will include all RCTs meeting our inclusion criteria irrespectively of the outcome(s) reported in the publication. The authors will thus be contacted to ask for information and the IPD for all outcomes relevant to the review.

Data collection process

The first author, or sponsor, of each included trial will be approached and asked to supply anonymised IPD, metadata, and relevant documentation[33] (protocol and blank case report forms) from the respective trial, either through direct contact, or through data sharing portals such as clinicalstudydatarequest.com, where relevant.

Before starting the quantitative synthesis, we will conduct a set of standard quality and consistency checks on the provided data. This will involve cross-checks by re-analysing IPD against previously published results to highlight inconsistencies or possible errors. Any queries will be raised with the original trialists wherever possible. Data will be cleaned and standardised to allow pooling and subsequent analyses of the data.

Data items

The data requested from each clinical trial will include at least:

For peer review only

- Baseline characteristics – age; sex; ethnicity; eczema; height; weight; baseline severity; baseline Peak Expiratory Flow Rate (PEFR); Forced Expiratory Volume in one second (FEV_1). Where available, we will also collect other variables such as age at asthma diagnosis, asthma-related hospitalization and exacerbation history, allergic sensitisation, parental asthma, objective indicators of asthma such as bronchodilator reversibility or methacholine PC20.
- The date of randomisation and dates of follow-up visits/interval between randomisation and follow up.
- Treatment details including inhalation device and dose.
- Adherence data if available.
- Data for the review outcomes along with details of their definitions and measurement tools used – symptoms; exacerbations; asthma control; mortality; quality of life; growth; physiological outcomes: Peak Expiratory Flow Rate (PEFR) and Forced Expiratory Volume in one second (FEV_1); hospital admissions.
- Cost, resource use, and utility outcomes to inform the economic model.

We will use data from the intention-to-treat (ITT) patients.

Outcomes and prioritisation

Primary outcomes

The primary outcomes of the study are exacerbations and loss of/change in asthma control measured by a validated test.

1. Exacerbations are defined as in ERS/ATS,[24] namely "*events characterized by a change from the patient's previous status*". We will define exacerbations as every event requiring the use of oral corticosteroids (OCS), the need for unscheduled visits with general practitioners (GPs), or at the emergency department (ED), and hospitalisation.
2. Based on the definition reported in ERS/ATS,[24] asthma control corresponds to "*the extent to which the various manifestations of asthma have been reduced or removed by treatment*".

For peer review only

Exacerbations are expected to be an individual-level numerical variable (binary, ordinal, or count) or an individual-level time-to-event variable. Asthma control is expected to be an individual-level numerical variable (categorical) and will be measured by a validated test (e.g., ACT,[25] ACQ,[26-29], other). Table 2 represents a possible categorisation for asthma control outcome based on ACT/ACQ scores. A standardisation method coherently to ACT and ACQ will be applied for other kinds of test. After consultation with clinicians, we will decide if, for this outcome variable, it is worthier to consider the full longitudinal profile (multiple measurements) or to establish a clinically relevant time-point.

Table 2 A possible categorisation of asthma control outcome based on ACT/ACQ scores

Test	Asthma control	Total score
**ACT 4-11 (years)**	0 = poor control	score ≤19
	1 = good/total control	score = 20–27
**ACT 12+ (years)**	0 = poor control	score ≤19
	1 = good/total control	score = 20–25
**ACQ**	0 = poor control	score >1
	1 = good/total control	score ≤1
**Others**	0 = poor control<br>1 = good/total control	to be evaluated on an individual case by case basis

Note: if both ACT and ACQ are present, we will choose ACT as the best choice.

Secondary outcomes

The secondary outcomes of the study are as follows.

1. Symptoms/symptom score – The mode to report symptoms or symptom score may be different across studies. We will distinguish between daytime and night-time symptoms, whatever they are, also considering their intensity.
2. Quality of life – QoL may have been measured differently across the studies. We will use the most frequent scale as a model to transform the remaining data using, e.g., established conversion tables.
3. Mortality – This variable may be reported in different ways across studies. It will always be converted to a binary variable.

For peer review only

4. Physiological measurements – FEV_1 in L/s and PEFR%. After consulting clinicians, we will decide if considering the full longitudinal profile (multiple measures) or establishing a clinically relevant time-point.
5. Adverse events – These may differ across the studies. We will consider relevant AEs, such as cardiac and neuropsychiatric disorders, infections, pneumonia, behavioural difficulty, growth, and diabetes.
6. Hospital admissions – This variable may be reported in different ways across studies. We will look for evidence such as dates of hospitalisation, adverse events indicating admission occurred, and variables directly reporting hospitalisation, between the randomisation visit and the end of the follow-up.

Economic outcomes

7. The primary economic outcome will be the incremental cost per quality-adjusted life-year (QALY) gained. This will be estimated from data on:
   a. Hospital admissions
   b. Other items resource use, costs, and utilities – These will be based on purposive searches of the economic and medical literature.

Risk of bias in individual studies

Two independent reviewers will use the Cochrane Risk of Bias tool[34] to record risk of bias concerning randomisation method, allocation concealment, blinding, incomplete outcome data, and selective reporting. Disagreements will be resolved through discussion with a third reviewer.

DATA

Synthesis

For every separate outcome, we will create a NMA diagram displaying the number of studies and patients for each treatment comparison within the network.

For peer review only

We will fit a Bayesian hierarchical meta-analysis model – supplemented with aggregate data if necessary – to synthesise the available IPD. We will thus estimate the relative treatment effect – odds ratio (OR) for categorical data and difference in means for continuous data – and credibility interval for every pair-wise comparison based on direct evidence. We will assess the homogeneity assumption by comparing the Device Information Criterion (DIC) of fixed-effect and random-effect models and observing the between trial variance. The forest plots, chi-square test for heterogeneity, and I^2 statistic will be examined to assess the evidence of heterogeneity within each pair-wise meta-analysis based on direct evidence.

We will conduct a NMA for every outcome within a Bayesian framework with the WinBUGS software with the Goodness-of-fit assessed by calculating the posterior mean residual deviance with DIC used as a basis for model comparison. Correlation between treatment effects from multi-arm trials will be appropriately accounted for. From the NMA, we can estimate the relative treatment effect for every pair-wise comparison irrespective of they have been compared directly in an RCT as well as calculating the probability that each treatment is the best, or worst, for a given outcome. In random-effects NMA models, we will check the conventional assumption that between-trial variance is the same for each comparison, by fitting pair-wise models based on direct evidence and assessing whether the variance is similar for each comparison. If the assumption appears unrealistic, we will explore other variance structures for the NMA model.

In a NMA, the indirect comparisons are not protected by randomisation, as for direct comparisons. So, they may be affected by confounding due to differences among clinical trials.[35] The validity of a NMA thus depends on the critical assumption of transitivity namely, to verify whether the probability of having been able to have given any of the treatment in the network to any patient in the network was equally likely.[35] We will check this assumption by comparing the inclusion/exclusion criteria of trials to make a judgement about whether patients, trial protocols, doses, administration procedures, and so on are similar in the modes that might modify treatment

For peer review only

effect. We will use model fit and selection statistics to informally assess whether inconsistency is evident along with a formal analysis using a 'node-splitting' approach.[36, 37]

We will explore potential patient-level characteristics that may modify treatment effects using hierarchical models with interaction effects between treatment and specific covariates. Initially, based on direct evidence, and subsequently in a NMA of the IPD, with aggregate data if IPD is unavailable.[38-41] The effect of covariates will be separated within and between trials. The underlying consistency assumption of these models will also be explored. Patient-level characteristics of interest include age, gender, ethnicity, eczema status, asthmatic phenotype, baseline %PEFR, and asthma severity. A literature search will be conducted to identify any further potential characteristics to explore.

We will fit a NMA model to compare compounds within each class and separate models to compare the different classes of treatments. Furthermore, complex hierarchical models that account for both classes and compounds with covariate interactions will be explored.

We will undertake sensitivity analyses for investigating the impact of missing IPD at the level of results and conclusions of NMA and for exploring the robustness of results to different priors in the Bayesian analyses. If sufficient data are available, sensitivity analyses that adjust for levels of adherence will be conducted to assess the robustness of results obtained from the primary analysis on the ITT patients.

For peer review only

Meta-bias(es)

To minimise the impact of selective outcome reporting, we will include all RCTs meeting our inclusion criteria irrespectively of the outcome(s) reported in the publication. For analyses containing 10 or more trials – due to the low power of the assessments for analyses containing small numbers of trials – we will investigate publication bias (and other selection bias/small study effects) by using funnel plots and appropriate statistical tests for funnel plot asymmetry.

Confidence in cumulative evidence

We will assess the quality of evidence for all outcomes across the domains of risk of bias, consistency, directness, precision, and publication bias according to GRADE (Grading of Recommendations Assessment, Development and Evaluation).

Economic analysis

The economic analysis will be conducted using standard methods,[42] based on the most robust data available and reported according to the CHEERS statement.[43] Cost-effectiveness will be estimated from the perspective of the NHS and Personal Social Services in line with NICE guidance[44] and based on an economic model that considers health outcomes, resource use, costs, and health utilities to estimate the incremental cost per quality-adjusted life-year (QALY) gained of each treatment. This analysis will not consider missed school or work.

The parameters needed to populate the models will include outcomes estimated by the NMA of the IPD (e.g., efficacy and safety parameters) and other parameters (e.g., utilities, resource use, and the long-term costs of care) that will require searching of evidence beyond the studies included in the NMA. These will be sourced from a purposive review of the literature, and by using specialist databases (e.g., NHS EED for resource use parameters, and the Tufts CEA registry for utility parameters) where necessary. Unit cost data will be derived from standard national sources (e.g., NHS reference costs[45] and the British National Formulary[46]).

A Markov model will be necessary for longer term extrapolation of costs and outcomes, to allow for any differential impacts of treatments over time, and for transition of patients among the 5 steps of treatment.[47] Results of the NMA will be converted to transition probabilities based on standard methods.[48, 49] The model will evaluate costs and outcomes over the lifetime of the patient cohorts: costs and benefits in future years will be discounted at an annual rate of 3.5% and varied between 0% and 6% in sensitivity analysis. Results from the model will be reported as incremental cost per QALY gained (ICERs) and compared with the NICE threshold range of £20,000 to £30,000 per QALY.

For peer review only

444 Uncertainties in all parameter inputs will be accounted for in the analysis by including parametric distributions for each point estimate, supplemented by expert opinion, where the evidence is not sufficiently detailed. This will enable probabilistic sensitivity analyses to be performed based on sampling from distributions using Monte Carlo simulation. The NMA estimates relative effects jointly, and the full joint distribution of these effects will be used in the economic model in order to preserve correlation.[50] Uncertainty in the optimally cost-effective treatment will be represented by cost-effectiveness acceptability curves (CEACs),[51] which present the probability that each drug is the most cost-effective at a given threshold of cost effectiveness. Where the IPD-NMA indicates that patient characteristics modify treatment effects, a stratified approach will be used to assess cost-effectiveness in particular patient sub-group(s). Standard techniques (e.g., extreme value scenarios) will be used to ensure the internal validity of the model.

We will conduct a value of information analysis to inform future research priorities, e.g., whether more short-term efficacy trials are needed, or more long-term follow-up, or more data on the utilities or costs to reduce decision uncertainty.[52] The expected value of perfect information (EVPI) and the expected value of perfect parameter information (EVPPI) will be calculated on both per-patient and population levels using the Sheffield Accelerated Value of Information approximation[53] to facilitate computation effort.

Ethics and dissemination

The University of Liverpool committee on Research Ethics has confirmed that ethics review is not required. The dissemination plan consists of publishing the results in an open-access medical journal, a plain-language summary available for parents and children, dissemination via local, national, and international meetings and conferences, and the Press Offices of our HEIs. A synopsis of results will be disseminated to NICE and BTS/SIGN as highly relevant to future clinical guideline updates.

Patient and Public Involvement

For peer review only

The EINSTEIN protocol has been developed in consultation with children with asthma and their parents, and with NHS clinicians who routinely care for children with uncontrolled asthma in NHS settings. Firstly, we have sought advice on our proposal, and the lay summary, from five families, including two children, who attend our asthma clinic at Alder Hey. Secondly, the outcomes chosen in our review have been selected from a core outcome set which were agreed as important by clinicians and patients.[21] This earlier work was heavily influenced by patients and, in particular, highlighted the importance to parents of including mortality and long-term adverse effects as outcomes. Finally, we consulted an Alder Hey patient advisory group, comprising children with asthma and their parents, and asked them to comment specifically on the lay summary and choice of outcomes in the review project. We will consult with the patient group towards the end of the project to help prepare a patient friendly podcast or leaflet that could be shared on asthma websites and in clinics. We will share our findings at important international meetings, and in medical journals, and we will make sure that this process reflects issues that are important to children and families. Our patient representative (OF) will help to coordinate the group of patients and parents, along with physicians involved. Patients will not be involved in the recruitment to and conduct of the study.

For peer review only

Authors' contributions

CTS is the guarantor. CTS and IS conceived the study. CTS, SD, ST, DH, OF and IS drafted the original version of the protocol, and SC drafted this manuscript. All authors contributed to the development of the selection and data extraction criteria and the strategy of risk of bias assessment. MM developed the search strategy. CTS and SD provided statistical expertise. IS and ST provided clinical expertise. KR and SC screened and selected eligible studies for inclusion in the review. DH and GC contributed to the section on health economics. OF contributed to coordinating the group of patients and parents and developing the plain-language summary. All authors read, provided feedback, and approved the final manuscript.

Funding statement

This work was supported by NIHR HTA grant number 16/110/16. The funder had no role in the protocol development. The views expressed are those of the authors and not necessarily those of the NHS, the NIHR or the Department of Health.

Competing interests statement

None declared.

Acknowledgements

We thank patients and their families for their contribution to this project.

REFERENCES

1 Global Initiative for Asthma (GINA). Global Strategy for Asthma Management and Prevention, 2019. www.ginasthma.org

2 Asthma UK. Access date: February 2020. https://www.asthma.org.uk/about/media/facts-and-statistics/

3 NHS England. Access date: February 2020. https://www.england.nhs.uk/childhood-asthma/

4 British Thoracic Society SIGN. British guideline on the management of asthma. Access Date: February 2020. https://www.brit-thoracic.org.uk/quality-improvement/guidelines/asthma/

5 Asthma: diagnosis, monitoring and chronic asthma management. Access Date: April 2020. https://www.nice.org.uk/guidance/ng80

6 Turner S, Thomas M, von Ziegenweidt J, et al. Prescribing trends in asthma: a longitudinal observational study. *Arch Dis Child* 2009;94(1):16–22. http://dx.doi.org/10.1136/adc.2008.140681

7 Schmier JK, Manjunath R, Halpern MT, et al. The impact of inadequately controlled asthma in urban children on quality of life and productivity. *Ann Allergy Asthma Immunol* 2007;98(3):245–51. https://doi.org/10.1016/S1081-1206(10)60713-2

For peer review only

8 Fuhrman C, Dubus JC, Marguet C, et al. Hospitalizations for asthma in children are linked to undertreatment and insufficient asthma education. *J Asthma* 2011;48(6):565–71. https://doi.org/10.3109/02770903.2011.580031

9 Sala KA, Carroll CL, Tang YS, et al. Factors associated with the development of severe asthma exacerbations in children. *J Asthma* 2011;48(6):558–64. https://doi.org/10.3109/02770903.2011.585411

10 Ungar WJ, Paterson JM, Gomes T, et al. Relationship of asthma management, socioeconomic status, and medication insurance characteristics to exacerbation frequency in children with asthma. *Ann Allergy Asthma Immunol* 2011;106(1):17–23. https://doi.org/10.1016/j.anai.2010.10.006

11 Stelmach I, Podlecka D, Smejda K, et al. Pediatric asthma caregiver's quality of life questionnaire is a useful tool for monitoring asthma in children. *Qual Life Res* 2012;21(9):1639–1642. https://doi.org/10.1007/s11136-011-0070-x

12 Milton B, Whitehead M, Holland P, et al. The social and economic consequences of childhood asthma across the lifecourse: a systematic review. *Child Care Health Dev* 2004;30(6):711–28. https://doi.org/10.1111/j.1365-2214.2004.00486.x

13 Szefler SJ, Phillips BR, Martinez FD, et al. Characterization of within-subject responses to fluticasone and montelukast in childhood asthma. *J Allergy Clin Immunol* 2005;115(2):233–42. https://doi.org/10.1016/j.jaci.2004.11.014

14 Lemanske RF, Mauger DT, Sorkness CA, et al. Step-up Therapy for Children with Uncontrolled Asthma While Receiving Inhaled Corticosteroids. *N Engl J Med* 2010;362(11):975–85. https://doi.org/10.1056/NEJMoa1001278

15 Chauhan BF, Chartrand C, Ni Chroinin M, et al. Addition of long-acting beta2-agonists to inhaled corticosteroids for chronic asthma in children. *Cochrane Database Syst Rev* 2015(11):CD007949. https://doi.org/10.1002/14651858.CD007949.pub2

For peer review only

16 Chauhan BF, Ben Salah R, Ducharme FM. Addition of anti-leukotriene agents to inhaled corticosteroids in children with persistent asthma. *Cochrane Database Syst Rev* 2013(10):CD009585. https://doi.org/10.1002/14651858.CD009585.pub2

17 Zhao Y, Han S, Shang J, et al. Effectiveness of drug treatment strategies to prevent asthma exacerbations and increase symptom-free days in asthmatic children: a network meta-analysis. *J Asthma* 2015;52(8):846–57. https://doi.org/10.3109/02770903.2015.1014101

18 van der Mark LB, Lyklema PH, Geskus RB, et al. A systematic review with attempted network meta-analysis of asthma therapy recommended for five to eighteen year olds in GINA steps three and four. *BMC Pulm Med* 2012;12:63. https://doi.org/10.1186/1471-2466-12-63

19 Hutton J, Williamson P. Bias in meta-analysis with variable selection within studies. *Applied Statistics* 2000;49:359–70. https://doi.org/10.1111/1467-9876.00197

20 Lenney W, McKay AJ, Tudur Smith C, et al. Management of Asthma in School age Children On Therapy (MASCOT): a randomised, double-blind, placebo-controlled, parallel study of efficacy and safety. *Health Technol Assess* 2013;17(4):1–218. https://doi.org/10.3310/hta17040

21 Lenney W, Perry S, Price D. Clinical trials and tribulations: the MASCOT study. *Thorax* 2011;66(6):457–8. https://doi.org/10.1136/thx.2010.156885

22 Moher D, Shamseer L, Clarke M, et al. Preferred reporting items for systematic review and meta-analysis protocols (PRISMA-P) 2015 statement. *Syst rev* 2015;4:1. https://doi.org/10.1186/2046-4053-4-1

23 Sinha IP, Gallagher R, Williamson PR, et al. Development of a core outcome set for clinical trials in childhood asthma: a survey of clinicians, parents, and young people. *Trials* 2012;13:103. https://doi.org/10.1186/1745-6215-13-103

24 Reddel HK, Taylor DR, Bateman ED, et al. An official American Thoracic Society/European Respiratory Society statement: asthma control and exacerbations: standardizing endpoints for clinical asthma trials and clinical practice. *Am J Respir Crit Care Med* 2009;180(1):59–99. https://doi.org/10.1164/rccm.200801-060ST

For peer review only

25 Nathan RA, Sorkness CA, Kosinski M, et al. Development of the asthma control test: a survey for assessing asthma control. *J Allergy Clin Immunol* 2004;113(1):59–65. https://doi.org/10.1016/j.jaci.2003.09.008

26 Juniper EF, O'Byrne PM, Guyatt GH, et al. Development and validation of a questionnaire to measure asthma control. *Eur Respir J* 1999;14:902–7. https://doi.org/10.1034/j.1399-3003.1999.14d29.x

27 Juniper EF, Svensson K, Mörk AC, et al. Measurement properties and interpretation of three shortened versions of the asthma control questionnaire. *Respir Med* 2005;99:553–8. https://doi.org/10.1016/j.rmed.2004.10.008

28 Juniper EF, Bousquet J, Abetz L, et al. Identifying 'well-controlled' and 'not well-controlled' asthma using the Asthma Control Questionnaire. *Respir Med* 2006;100:616–621. https://doi.org/10.1016/j.rmed.2005.08.012

29 Juniper EF, Gruffydd-Jones K, Ward S, et al. Asthma Control Questionnaire in Children: Validation, Measurement Properties, Interpretation. *Eur Respir J* 2010;36:1410–1416. https://doi.org/10.1183/09031936.00117509

30 Chauhan BF, Ducharme FM. Addition to inhaled corticosteroids of long-acting beta2-agonists versus anti-leukotrienes for chronic asthma. *Cochrane Database Syst Rev* 2014(1):CD003137. https://doi.org/10.1002/14651858.CD003137.pub5

31 Chauhan BF, Ducharme FM. Anti-leukotriene agents compared to inhaled corticosteroids in the management of recurrent and/or chronic asthma in adults and children. *Cochrane Database Syst Rev* 2012(5):CD002314. https://doi.org/10.1002/14651858.CD002314.pub3

32 Pruteanu AI, Chauhan BF, Zhang L, et al. Inhaled corticosteroids in children with persistent asthma: dose-response effects on growth. *Cochrane Database Syst Rev* 2014(7):CD009878. https://doi.org/10.1002/14651858.CD009878.pub2

For peer review only

33 Tudur Smith C, Hopkins C, Sydes MR, et al. How should individual participant data (IPD) from publicly funded clinical trials be shared? *BMC Med* 2015;13(1):1–7. https://doi.org/10.1186/s12916-015-0532-z

34 Higgins JPT, Altman DG, Gøtzsche PC, et al. The Cochrane Collaboration's tool for assessing risk of bias in randomised trials. *BMJ* 2011;343:d5928. https://doi.org/10.1136/bmj.d5928

35 The transitivity assumption CJC. Available from: https://airways.cochrane.org/sites/airways.cochrane.org/files/public/uploads/The transitivity assumption CJC.pdf

36 Dias S, Welton NJ, Caldwell DM, et al. Checking consistency in mixed treatment comparison meta-analysis. *Stat Med* 2010;29(7-8):932–44. https://doi.org/10.1002/sim.3767

37 Donegan S, Williamson P, D'Alessandro U, et al. Assessing key assumptions of network meta-analysis: a review of methods. *Res Synth Methods* 2013;4(4):291–323. https://doi.org/10.1002/jrsm.1085

38 Donegan S, Williamson P, D'Alessandro U, et al. Combining individual patient data and aggregate data in mixed treatment comparison meta-analysis: Individual patient data may be beneficial if only for a subset of trials. *Stat Med* 2013;32(6):914–30. https://doi.org/10.1002/sim.5584

39 Cooper NJ, Sutton AJ, Morris D, et al. Addressing between-study heterogeneity and inconsistency in mixed treatment comparisons: Application to stroke prevention treatments in individuals with non-rheumatic atrial fibrillation. *Stat Med* 2009;28(14):1861–81. https://doi.org/10.1002/sim.3594

40 Saramago P, Sutton AJ, Cooper NJ, et al. Mixed treatment comparisons using aggregate and individual participant level data. *Stat Med* 2012;31(28):3516–36. https://doi.org/10.1002/sim.5442

41 Donegan S, Williamson P, D'Alessandro U, et al. Assessing the consistency assumption by exploring treatment by covariate interactions in mixed treatment comparison meta-analysis:

For peer review only

individual patient-level covariates versus aggregate trial-level covariates. *Stat Med* 2012;31(29):3840–57. https://doi.org/10.1002/sim.5470

42 Petrou S, Gray A. Economic evaluation using decision analytical modelling: design, conduct, analysis, and reporting. *BMJ* 2011;342:d1766. https://doi.org/10.1136/bmj.d1766

43 Husereau D, Drummond M, Petrou S, et al. Consolidated Health Economic Evaluation Reporting Standards (CHEERS) statement. *BMJ* 2013;346:f1049. https://doi.org/10.1136/bmj.f1049

44 National Institute for Health and Care Excellence. Guide to the methods of technology appraisal 2013. Available from: https://www.nice.org.uk/process/pmg9

45 NHS Improvement. NHS Reference costs 2017/18. https://improvement.nhs.uk/documents/6468/201718_reference_costs_data_and_guidance.zip

46 British National Formulary 79. March-September 2020

47 Ehteshami-Afshar S, Zafari Z, Hamidi N, et al. A Systematic Review of Decision-Analytic Models for Evaluating Cost-Effectiveness of Asthma Interventions. *Value Health* 2019;22(9):1070–1082. https://doi.org/10.1016/j.jval.2019.03.016

48 Gidwani R, Russell LB. Estimating Transition Probabilities from Published Evidence: A Tutorial for Decision Modelers. *Pharmacoeconomics* 2020;38(11):1153–1164. https://doi.org/10.1007/s40273-020-00937-z

49 Welton NJ, Sutton AJ, Cooper NJ, et al. Evidence Synthesis for Decision Making in Healthcare, John Wiley & Sons, Ltd. 2012.

50 Dias S, Sutton AJ, Welton NJ, et al. Evidence synthesis for decision making 6: embedding evidence synthesis in probabilistic cost-effectiveness analysis. *Med Decis Making* 2013;33(5):671-8. https://doi.org/10.1177/0272989X13487257

51 Fenwick E, Claxton K, Sculpher M. Representing uncertainty: the role of cost-effectiveness acceptability curves. *Health Econ* 2001;10(8):779-87. https://doi.org/10.1002/hec.635

For peer review only

52 Claxton KP, Sculpher MJ. Using value of information analysis to prioritise health research: some lessons from recent UK experience. *Pharmacoeconomics* 2006;24(11):1055-68. https://doi.org/10.2165/00019053-200624110-00003

53 Strong M, Oakley JE, Brennan A. Estimating multi-parameter partial expected value of perfect information from a probabilistic sensitivity analysis sample: a non-parametric regression approach. *Med Decis Making* 2014;34(3):311-26. https://doi.org/10.1177/0272989X13505910

Figure caption

Figure 1 Treatment step-up for asthmatic patients according to age group. Adapted from GINA 2019.[1] SABA = Short-acting β_2-adrenoceptor agonist; ICS = Inhaled corticosteroid; LTRA = Leukotriene receptor antagonist; LABA = Long-acting β_2-adrenoceptor agonist; OCS = Oral corticosteroids ; IgE = Immunoglobulin E; IL = Interleukin

For peer review only

≤ 5-year-old (Children)

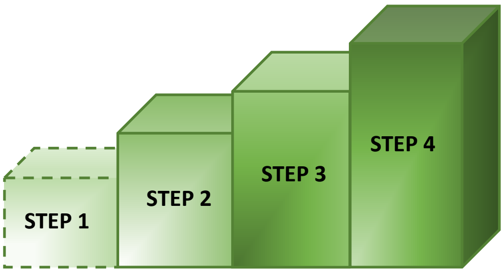

Reliever: as-needed SABA

STEP 1 SABA as needed
STEP 2 Daily low dose ICS – SABA as needed.
Other controller options: LTRA, or intermittent ICS.
STEP 3 Double low dose ICS – SABA as needed
Other controller options: low dose ICS + LTRA.
STEP 4 Continue controller + specialist assessment
Other controller options: add LTRA / increase ICS frequency / add intermittent ICS.

6-11-year-old (Children)

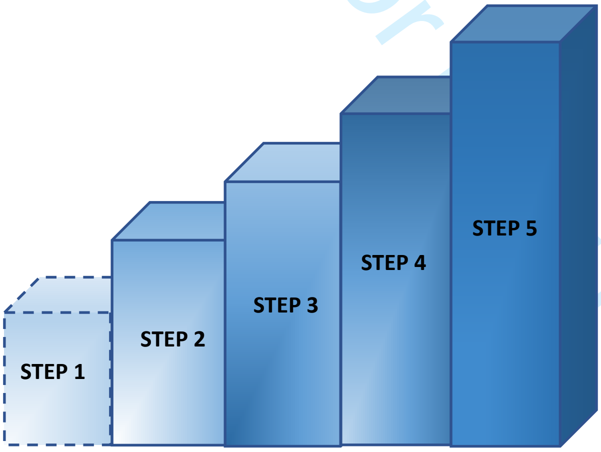

Reliever: as-needed SABA

STEP 1 SABA as needed.
Other controller options: low dose of ICS taken with SABA, or daily low dose ICS.
STEP 2 Daily low dose ICS – SABA as needed.
Other controller options: LTRA, or low dose ICS taken with SABA.
STEP 3 Low dose ICS+LABA, or medium dose ICS – SABA as needed. *Other controller options*: low dose ICS+LTRA.
STEP 4 Medium dose ICS+LABA – SABA as needed.
Other controller options: high dose ICS+LABA, or add-on tiotropium, or add-on LTRA.
STEP 5 Phenotypic assessment ± add-on therapy (e.g., anti-IgE) – SABA as needed. *Other controller options*: add-on anti-IL5, or add-on low dose OCS.

≥ 12-year-old (Adults and adolescents)

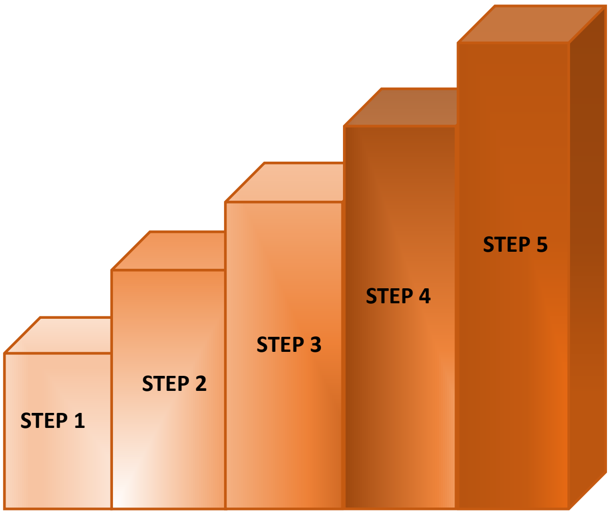

Preferred reliever: as-needed low dose ICS/formoterol
Reliever option: as-needed SABA

STEP 1 Low dose ICS/formoterol as needed.
Other controller options: low dose of ICS taken with SABA.
STEP 2 Daily low dose ICS – Low dose ICS/formoterol as needed. *Other controller options*: LTRA, or low dose ICS taken with SABA.
STEP 3 Low dose ICS+LABA – Low dose ICS/Formoterol as needed. *Other controller options*: medium dose ICS, or low dose ICS+LTRA.
STEP 4 Medium dose ICS+LABA – Low dose ICS/Formoterol as needed. *Other controller options*: high dose ICS, add-on tiotropium, or add-on LTRA.
STEP 5 High dose ICS+LABA. Phenotypic assessment ± add-on therapy (e.g., tiotropium, anti-IgE, anti-IL5/5R, anti-IL4R) – Low dose ICS/Formoterol as needed.
Other controller options: add-on low dose OCS.

EstablishINg the best STEp-up treatments for children with uncontrolled asthma despite INhaled corticosteroids (EINSTEIN): Protocol for a systematic review, network meta-analysis and cost effectiveness analysis using individual participant data (IPD)

Sofia Cividini[1], Ian Sinha[2], Sarah Donegan[1], Michelle Maden[3], Giovanna Culeddu[4], Katie Rose[2], Olive Fulton[*], Dyfrig A. Hughes[4], Stephen Turner[5], Catrin Tudur Smith[1]

[1]Department of Biostatistics, University of Liverpool, Liverpool, UK
[2]Alder Hey Children's Foundation NHS Trust, Liverpool, UK
[3]Liverpool Reviews and Implementation Group (LRIG), University of Liverpool, Liverpool, UK
[4]Centre for Health Economics & Medicines Evaluation, Bangor University, Bangor, UK
[5]University Court of the University of Aberdeen, Aberdeen, UK
[*]Patients Representative, Liverpool, UK

For peer review only

Supplementary File 1. Example MEDLINE (OVID) search

1 exp Asthma/
2 asthma.ti,ab.
3 1 or 2
4 exp Infant/
5 infant*.ti,ab.
6 infancy.ti,ab.
7 newborn*.ti,ab.
8 baby*.ti,ab.
9 babies.ti,ab.
10 neonat*.ti,ab.
11 preterm*.ti,ab.
12 prematur*.ti,ab.
13 postmatur*.ti,ab.
14 exp child/
15 child*.ti,ab.
16 schoolchild*.ti,ab.
17 "school age*".ti,ab.
18 preschool*.ti,ab.
19 kid.ti,ab.
20 kids.ti,ab.
21 toddler*.ti,ab.
22 exp Adolescent/
23 adoles*.ti,ab.
24 teen*.ti,ab.
25 boy*.ti,ab.
26 girl*.ti,ab.
27 exp Minors/
28 minor*.ti,ab.
29 exp Puberty/
30 pubert*.ti,ab.
31 pubescen*.ti,ab.
32 prepubescen*.ti,ab.
33 exp Pediatrics/
34 paediatric*.ti,ab.
35 pediatric*.ti,ab.

2

36 exp Schools/
37 "nursery school*".ti,ab.
38 kindergar*.ti,ab.
39 "primary school*".ti,ab.
40 "secondary school*".ti,ab.
41 "elementary school*".ti,ab.
42 "high school*".ti,ab.
43 highschool*.ti,ab.
44 or/4-43
45 "inhaled corticosteroid*".mp.
46 ICS.mp.
47 exp Beclomethasone/
48 beclomethasone.mp.
49 "beclomethasone dipropionate".mp.
50 becotide.mp.
51 clenil.mp.
52 ciclesonide.mp.
53 "clenil modulite".mp.
54 exp Fluticasone/
55 "fluticasone propionate".mp.
56 fluticasone.mp.
57 flixotide.mp.
58 exp Budesonide/
59 budesonide.mp.
60 Mometasone Furoate/
61 mometasone.mp.
62 exp Adrenergic beta-Agonists/
63 "long acting beta-2 agonist*".mp.
64 "long acting beta2 agonist*".mp.
65 LABA.mp.
66 exp Formoterol Fumarate/
67 formoterol.mp.
68 Oxis.mp.
69 "fluticasone furoate".mp.
70 exp Salmeterol Xinafoate/
71 salmeterol.mp.
72 serevent.mp.
73 vilanterol.mp.
74 exp Leukotriene Antagonists/
75 "leukotriene receptor antagonist*".mp.
76 LTRA.mp.
77 zafirlukast.mp.
78 montelukast.mp.
79 exp Theophylline/
80 theophylline.mp.
81 Tiotropium.mp.
82 spiriva.mp.
83 Symbicort.mp.
84 Seretide.mp.
85 flutiform.mp.
86 relvar.mp.
87 or/45-86
88 Clinical Trial.pt.

For peer review only

89 Randomized Controlled Trial.pt.
90 exp Random Allocation/
91 exp Single-Blind Method/
92 exp Double-Blind Method/
93 exp Cross-Over Studies/
94 exp Placebos/
95 RCT.ti,ab.
96 Random*.ti,ab.
97 "Single blind*".ti,ab.
98 "Double blind*".ti,ab.
99 "triple blind*".ti,ab.
100 placebo*.ti,ab.
101 or/88-100
102 3 and 44 and 87 and 101
103 limit 102 to ed=20140701-20190911
104 limit 103 to english language
105 (case reports or editorial or letter).pt.
106 104 not 105

For peer review only

PRISMA-P 2015 Checklist

This checklist has been adapted for use with protocol submissions to *Systematic Reviews* from Table 3 in Moher D et al: Preferred reporting items for systematic review and meta-analysis protocols (PRISMA-P) 2015 statement. *Systematic Reviews* 2015 **4**:1

Section/topic	#	Checklist item	Information reported: Yes	Information reported: No	Line number(s)
**ADMINISTRATIVE INFORMATION**					
**Title**					
Identification	1a	Identify the report as a protocol of a systematic review	■	□	1-3
Update	1b	If the protocol is for an update of a previous systematic review, identify as such	□	□	
**Registration**	2	If registered, provide the name of the registry (e.g., PROSPERO) and registration number in the Abstract	■	□	67
**Authors**					
Contact	3a	Provide name, institutional affiliation, and e-mail address of all protocol authors; provide physical mailing address of corresponding author	■	□	6-23
Contributions	3b	Describe contributions of protocol authors and identify the guarantor of the review	■	□	479-486
**Amendments**	4	If the protocol represents an amendment of a previously completed or published protocol, identify as such and list changes; otherwise, state plan for documenting important protocol amendments	□	□	
**Support**					
Sources	5a	Indicate sources of financial or other support for the review	■	□	67-68<br>487-490
Sponsor	5b	Provide name for the review funder and/or sponsor	■	□	67-68<br>487-490
Role of sponsor/funder	5c	Describe roles of funder(s), sponsor(s), and/or institution(s), if any, in developing the protocol	■	□	487-490
**INTRODUCTION**					
**Rationale**	6	Describe the rationale for the review in the context of what is already known	■	□	83-165
**Objectives**	7	Provide an explicit statement of the question(s) the review will address with reference to participants, interventions, comparators, and outcomes (PICO)	■	□	166-186

BioMed Central
The Open Access Publisher

For peer review only

Section/topic	#	Checklist item	Information reported		Line number(s)
			Yes	No	
**METHODS**					
**Eligibility criteria**	8	Specify the study characteristics (e.g., PICO, study design, setting, time frame) and report characteristics (e.g., years considered, language, publication status) to be used as criteria for eligibility for the review	■	□	190-258
**Information sources**	9	Describe all intended information sources (e.g., electronic databases, contact with study authors, trial registers, or other grey literature sources) with planned dates of coverage	■	□	260-278
**Search strategy**	10	Present draft of search strategy to be used for at least one electronic database, including planned limits, such that it could be repeated	■	□	260-278
STUDY RECORDS					
Data management	11a	Describe the mechanism(s) that will be used to manage records and data throughout the review	■	□	281-282
Selection process	11b	State the process that will be used for selecting studies (e.g., two independent reviewers) through each phase of the review (i.e., screening, eligibility, and inclusion in meta-analysis)	■	□	284-288
Data collection process	11c	Describe planned method of extracting data from reports (e.g., piloting forms, done independently, in duplicate), any processes for obtaining and confirming data from investigators	■	□	290-298
**Data items**	12	List and define all variables for which data will be sought (e.g., PICO items, funding sources), any pre-planned data assumptions and simplifications	■	□	300-315
**Outcomes and prioritization**	13	List and define all outcomes for which data will be sought, including prioritization of main and additional outcomes, with rationale	■	□	317-361
**Risk of bias in individual studies**	14	Describe anticipated methods for assessing risk of bias of individual studies, including whether this will be done at the outcome or study level, or both; state how this information will be used in data synthesis	■	□	363-365
DATA					
**Synthesis**	15a	Describe criteria under which study data will be quantitatively synthesized	■	□	368-412
	15b	If data are appropriate for quantitative synthesis, describe planned summary measures, methods of handling data, and methods of combining data from studies, including any planned exploration of consistency (e.g., I^2, Kendall's tau)	■	□	368-412
	15c	Describe any proposed additional analyses (e.g., sensitivity or subgroup analyses, meta-regression)	■	□	368-412
	15d	If quantitative synthesis is not appropriate, describe the type of summary planned	■	□	368-412
**Meta-bias(es)**	16	Specify any planned assessment of meta-bias(es) (e.g., publication bias across studies, selective	■	□	414-418

For peer review only

BioMed Central The Open Access Publisher

Section/topic	#	Checklist item	Information reported		Line number(s)
			Yes	No	
		reporting within studies)			
**Confidence in cumulative evidence**	17	Describe how the strength of the body of evidence will be assessed (e.g., GRADE)	■	☐	420-422

For peer review only

BioMed Central
The Open Access Publisher
